# Remembered Event Features Shape Default Mode Network Engagement during Emotional Memory Recall

**DOI:** 10.1523/ENEURO.0146-26.2026

**Published:** 2026-11-06

**Authors:** Nina Curko, Rosalie Samide, Valentina Krenz, Elizabeth A. Kensinger, Maureen Ritchey

**Affiliations:** ^1^Department of Psychology and Neuroscience, Boston College, Chestnut Hill, Massachusetts 02467; ^2^Department of Psychology, University of California, Los Angeles, California 90095

**Keywords:** default mode network, emotional memory, episodic memory, fMRI

## Abstract

Episodic memory involves reconstruction of past events through integration of multiple types of information, including perceptual details, narrative content, and emotional tone. The default mode network (DMN) is a set of regions thought to support episodic retrieval, yet it remains unclear how distinct subnetworks contribute to recall of these different memory features. Here, we examined how DMN subnetworks support and represent memory for naturalistic emotional events. Male and female human participants encoded short news videos that varied in emotional valence and recalled them in response to neutral cues during functional MRI scanning. Videos were again recalled 1 d later and memories were scored for perceptual and narrative details. Activity in the dorsomedial subnetwork was related to the emotional valence of the memory, while activity in the medial temporal subnetwork was associated with the number of perceptual details recalled. Multivariate pattern analyses further revealed that the medial temporal subnetwork exhibited greater pattern stability across recalls when recalling more perceptual details, while stability in the dorsomedial and core subnetworks was tied to emotional remembering. Our findings suggest that the dorsomedial subnetwork provides an affective frame for a memory, while the medial temporal subnetwork contributes perceptual specificity. These results demonstrate that the contents of memory retrieval shape network engagement during emotional recall, providing insight into how the brain reconstructs complex real-world experiences.

## Significance Statement

How do we remember the emotional tone of an event versus its visual details? This study examines how distinct subnetworks within the brain's default mode network (DMN) contribute to remembering different features of memory. While the medial temporal subnetwork is connected to retrieving the perceptual details of past events, the dorsomedial subnetwork supports recall of the emotional tone. Furthermore, patterns of activity in the medial temporal subnetwork are more stable when recalling more perceptually rich memories, while stability in the dorsomedial subnetwork is tied to emotional remembering. These findings suggest that the DMN flexibly responds to different kinds of memory features, supporting the reconstruction of rich emotional memories from complex real-world experiences.

## Introduction

Episodic memory retrieval involves reconstructing multiple event features into a cohesive representation. This process is thought to be supported by the default mode network (DMN), a set of interconnected regions active during internal cognitive processes such as memory retrieval, episodic construction, and emotional experiences (Buckner et al., 2008; Andrews-Hanna et al., 2010; Rugg and Vilberg, 2013; Satpute and Lindquist, 2019). Emerging evidence suggests the DMN represents and integrates event-specific information in memory (Ritchey and Cooper, 2020). A major factor affecting how event features contribute to the experience of memory is the presence of positive or negative emotional content (Williams et al., 2022). However, it remains unclear whether different DMN components contribute to remembering distinct event features and, in particular, how DMN engagement during recall is shaped by the emotionality of memory.

The DMN comprises functionally distinct subnetworks, including a medial temporal subsystem associated with episodic processes and a dorsomedial subsystem associated with social cognition (Andrews-Hanna et al., 2010). Within the context of memory, these subnetworks may be differently related to the content of event representations, with regions in the medial temporal subsystem supporting memory for spatial and event context (Ranganath and Ritchey, 2012) and regions in the dorsomedial subsystem supporting representations of objects and their social and emotional associations (Barnett et al., 2021; Karagoz et al., 2023). These networks are differentially recruited when people orient toward the perceptual details and conceptual themes of memory (Gurguryan and Sheldon, 2019; Ferris et al., 2025). These frameworks suggest flexible recruitment of these subnetworks depending on memory content and, specifically, the perceptual and narrative richness of recall.

Another open question is how DMN subnetworks contribute to emotional memories. Emotional content biases memory toward central event details (Kensinger et al., 2007), facilitating abstraction of an event's high-level meaning. This abstraction process has been attributed to the dorsomedial prefrontal cortex (Baetens et al., 2014), implicating this region—and its associated DMN subnetwork—in integrating an event's affective tone (Kensinger and Ford, 2021). For real-world memories, both perceptual and narrative details may contribute to affective experience; however, they may be differently weighted for negative and positive valenced memories, with stronger engagement of perceptual regions during negative memory retrieval (Erk et al., 2003; Markowitsch et al., 2003; Mickley and Kensinger, 2008). Together, these findings suggest that emotion modulates how DMN subnetworks contribute to the reconstruction of different event details, but this has not been explicitly tested.

To address these questions, this study aims to characterize the role of DMN subnetworks in retrieving the details of emotional and neutral memories. Video stimuli were used to promote rich, varied recall of life-like events. Participants encoded short news clips varying in emotional valence (Samide et al., 2020) and recalled them during fMRI scanning. Event memories persisting 1 d later were scored for narrative and perceptual details. We hypothesized that medial temporal subnetwork activity would be associated with perceptual detail memory, whereas dorsomedial and core midline subnetworks would relate to narrative detail memory. We further hypothesized that dorsomedial network activity would be modulated by emotional content and that sensitivity to perceptual detail in the medial temporal network would be enhanced by negative valence.

In addition to univariate activity changes, emerging evidence indicates that activity patterns in DMN areas are sensitive to the specific details of events (Baldassano et al., 2017; Chen et al., 2017; Kuhl and Chun, 2014) and that pattern reinstatement during recall scales with how many details are remembered (Bird et al., 2015; Oedekoven et al., 2017). Less is known, however, about how these patterns correspond with the type of information remembered. One possibility is that event-specific recall patterns are influenced by narrative details that provide a consistent event model; alternatively, they might reflect recapitulation of perceptual details. To test these possibilities, we applied multivariate pattern analyses to examine whether DMN subnetworks represented event-specific details and whether the strength and stability of these representations related to the persistence of narrative and perceptual details in memory.

## Materials and Methods

### Participants

This study recruited participants (*N* = 35, 20 females, 15 males) from the Greater Boston area, with an age range of 18–35 (*M* = 22.29). Participants were right-handed, spoke English fluently, and indicated no history of psychiatric or neurological conditions and no use of psychoactive medications. Two subjects were excluded due to excessive head motion, two subjects were excluded because they exited the scanner early due to discomfort, and one subject was excluded due to a computer error during their session, leading to a final sample of 30 (16 females, 14 males) participants. All participants provided informed consent as approved by the Boston College Institutional Review Board and completed MRI and COVID safety screenings in compliance with Boston University Cognitive Neuroimaging Center protocols.

### Design

#### Encoding task

Before entering the scanner, participants first watched 48 clips of real-life news broadcasts of varying emotional valences (24 negative, 12 positive, 12 neutral) from the Samide et al. (2020) stimulus set (Fig. 1*a*). Video clips were randomly presented, ranged from 20 to 52 s in length, and lasted 42 s on average. Each video showed a news report focused on one particular topic, such as a soldier homecoming, a weather report, or a murder. During each video, a semantically related, affectively neutral cue word was simultaneously displayed on the screen. For example, a video about a school chemistry lab fire used the cue word “chemistry.” Participants were informed they would be asked to recall the video from the cue word later. After each video, participants rated the valence of the clip, i.e., how emotionally negative or positive they found the clip, on a nine-point scale from 1 (most negative) to 9 (most positive) using buttons in the scanner. They then rated the intensity of the clip, i.e., how “emotionally intense” they found the clip, from 1 (least intense) to 9 (most intense). Mean valence ratings differed significantly across normative categories, (*F*_(2,917)_ = 837.30, *p* < 0.001, all Tukey post hoc comparisons *p* < 0.001), with ratings increasing from negative (*M* = 2.85, SD = 1.54) to neutral (*M* = 5.95, SD = 1.35) to positive (*M* = 7.45, SD = 1.30; Fig. 1*c*). Participants were set up in the scanner immediately following the encoding phase.

#### Template task

In the scanner, participants first completed a “template” task. The purpose of this task was twofold: one, to reinforce the video–cue associations after the participants were in the scanner, and two, to provide a “template” of brain activity corresponding to each video's specific details, for comparisons with the later cued recall phase. Participants viewed a series of 10 chronological still images from each video (500 ms each × 10 = 5 s video period), with the relevant cue word displayed for 2 s after each video. In between trials, participants completed an active intertrial interval task (ITI) between each video in which they selected whether arrows appearing on the screen were pointing left or right. Each ITI period lasted 11 s. All video sets were shown once, divided across two functional runs.

#### Cued recall task

The cued recall task consisted of three periods for each trial: “access” (5 s), “elaboration” (10 s), and “success rating” (2 s). Participants were shown the previously assigned word cues and were instructed to silently recall the memory of the video associated with each cue. Participants pushed a button once they initially remembered the correct association. The “access” period lasted 5 s, regardless of when the button was pushed. After 5 s, a box appeared around the cue word to signal the 10 s “elaboration” period. In total, participants remembered each video for 15 s per trial. For 2 s at the end of each trial, participants were prompted to rate their success in recalling the memory using a scale ranging from 1 (not at all successful) to 5 (very successful). Between each video, participants completed the same ITI task as in the template phase, with a duration of 10 s.

Participants were cued to remember 12 negative, 12 positive, and 12 neutral videos for each round of the cued recall task. This cued recall task was repeated for a total of three rounds, with each cue being presented once per round. In other words, each video was recalled three times. Each round separated into three consecutive runs, resulting in a total of nine runs. The order of video presentation was randomized across participants and across rounds for each participant. Note that, for each participant, an additional 12 negative videos were recalled under instructions to reappraise the video during the elaboration period (four additional trials per run); these videos and the corresponding trials were retained in the dataset but excluded from the current analyses.

#### Delayed recall task

The next day, participants completed a memory test online, with experimenter instructions provided via Zoom call (Fig. 1*b*). Participants typed out their memories for each video associated with the corresponding cue word on the screen. Participants were instructed to provide as much detail as possible in describing their memory of the video and to include everything that came to mind in their description, such as storyline, setting, time, visual descriptions, thoughts, and feelings. Following each recall response, participants rated the vividness of the memory (1, least vivid; 9, most vivid), the valence of the memory (1, most negative; 9, most positive), and the emotional intensity of the memory (1, least intense; 9, most intense). In cases where participants could not remember the original video that matched the cue word, they were instructed to write “forgot” and enter “0” for the subsequent ratings. There was no time limit to complete each recall response. Each video was recalled once (48 total cues displayed).

### Scoring

Cued recall data were hand-scored by counting the occurrence of a detail belonging to one of the following categories, using a scheme derived from the Autobiographical Interview scoring protocol (Levine et al., 2002): event, visual, place, time, emotion, reaction, extra, and repeated, which were then further labeled as correct or incorrect. A detail was considered “correct” if it accurately referenced any part of the video. The three categories used in the following analyses are “event” (happenings, actions, or people that are involved in the storyline), “visual” (purely visual perceptual aspects not integral to the storyline), and “correct” (the total number of correct details reported from any category). All other scored categories did not yield high enough detail counts for analysis and thus were unsuitable for inclusion in the present study (category means across participants <1 detail). Two raters were trained on detail scoring until they achieved high reliability (correct detail inter-rater correlation, 0.92; narrative, 0.83; perceptual, 0.93); the remaining datasets were scored by one of these raters under the supervision of a graduate student. Here, narrative details are operationalized as event details, and perceptual details are operationalized as visual details.

### MRI data acquisition

MRI data were collected using a 3 T Siemens MAGNETOM Prisma MRI scanner with a 32-channel head coil at the Boston University Cognitive Neuroimaging Center. Structural MRI images were collected using a T1-weighted multiecho MPRAGE protocol [van der Kouwe et al., 2008; field of view, 256 mm; GRAPPA (iPAT) acceleration, 4 (Griswold et al., 2002); 1 mm isotropic voxels; 176 sagittal slices with interleaved acquisition; TR, 2,530 ms; TE, 1.69/3.55/5.41/7.27 ms; flip angle, 7°; anterior-to-posterior phase encoding]. Functional images were acquired using a whole-brain multiband (Moeller et al., 2010) echoplanar imaging (EPI) sequence (bandwidth, 1,718; field of view, 208 mm; 2 mm isotropic voxels; 69 slices with interleaved acquisition; TR, 1,500 ms; TE, 28 ms; flip angle, 75; anterior-to-posterior phase encoding with a simultaneous multislice factor, 3; for a total of 296 TRs per scan run). Field map scans were acquired at the beginning of the scan session, then again after the third cued recall run to correct EPI images for signal distortion.

### Preprocessing and modeling of fMRI data

MRI data were converted to NIfTI format using dcm2nii (Li et al., 2016) and converted to Brain Imaging Data Structure (BIDS) format using custom scripts in MATLAB (The Mathworks, 2019), verified using the BIDS validator (http://bids-standard.github.io/bids-validator/). MRIQC v0.15.1 (Esteban et al., 2017a) was used to assess data quality. Scan runs were excluded from analyses if >20% of time points exceeded a framewise displacement of 0.30 mm. Two participants were excluded because three of their scan runs met this criterion (final *N* = 30). An additional three participants were retained but had one or two of their runs excluded based on this criterion.

MRI data were preprocessed using fmriprep v1.5.2 with the default processing steps (Esteban et al., 2017b). The brain was extracted and segmented from T1w images, which were then normalized to MNI space. BOLD images were skull stripped, slice timing corrected, field map corrected, and registered to the T1w images. Data remained unsmoothed to minimize blurring of signals across region-of-interest (ROI) boundaries. Framewise displacement, six realignment parameters, and six aCompCor components derived from fmriprep were included as nuisance regressors in each model.

After preprocessing, data were entered into a model containing only the nuisance regressors listed above using SPM12. The residuals from this model were saved out in order to obtain corrected time series data. Residual activity was averaged across timepoints within a 15 s recall trial (a combination of the 5 s access and 10 s elaboration period, corrected for hemodynamic lag) to obtain individual voxel activity for each trial. Then, activity was averaged across all voxels in each parcel to obtain ROI activity for each trial. Trials were excluded from analysis if any ROI activity values were >4 standard deviations from the subject's mean (following Cooper and Ritchey, 2020). After all scan and trial exclusions, analyses were based on the following trial counts per participant: positive *M* = 32 (SD = 4.43); neutral *M* = 32.333 (SD = 3.96); and negative *M* = 29 (S*D* = 5.445).

### ROI definition

Neuroimaging data were extracted from the 200-parcel, 17-network cortical parcellations using the Schaefer-Yeo atlas (Schaefer et al., 2018). We targeted three default subnetworks: the core midline, the dorsomedial subnetwork, and the medial temporal subnetwork. Here, the core midline is delineated as DMN-A in the Schaefer-Yeo parcellation (Schaefer et al., 2018) and includes components of the medial prefrontal and parietal regions (anterior angular gyrus, posterior cingulate cortex). The dorsomedial subnetwork is referred to as DMN-B, including parcels in the dorsal and ventral prefrontal cortex and lateral temporal regions. The medial temporal subnetwork refers to medial temporal regions delineated as DMN-C and includes parcels in the parahippocampal cortex, ventral posterior cingulate, and posterior angular gyrus. To verify that these groupings were meaningful in our dataset, we examined within- and between-network parcel correlations and found that parcels from the same subnetwork were coactivated more strongly with each other during the task than with parcels from other subnetworks (Extended Data Fig. 2-1).

Four additional medial temporal lobe (MTL) ROIs were added for the left and right hippocampus and amygdala. The hippocampus was obtained from a probabilistic MTL atlas (Ritchey et al., 2015; https://neurovault.org/collections/3731/), and the amygdala was added from the Harvard–Oxford subcortical atlas, in line with prior methods (Cooper and Ritchey, 2019). Six additional hippocampal subfield ROIs (left and right head, body, and tail) were also used for secondary analyses (Ritchey et al., 2015; https://neurovault.org/collections/3731/), with body and tail collapsed to represent the posterior hippocampus.

### Statistical analysis

All statistical analyses were conducted using R 4.3.2 (https://www.r-project.org/). Custom code used to analyze this data can be found in the following repository: https://github.com/memobc/reframe-detail-paper. For the behavioral analyses, ANOVAs were used to examine the effect of emotional valence (positive, neutral, negative) on the average number of narrative, perceptual, and overall correct details recalled, using the R package *ez* (Lawrence, 2016); post hoc comparisons were computed using paired-sample *t* tests. For the fMRI analyses, time series-averaged trial activity in each network was predicted using linear mixed-effects models, which included the subject as a random intercept utilizing the R packages *lme4* (Bates et al., 2015) and *lmerTest* (Kuznetsova et al., 2017). We tested models including random slopes for valence and details; however, these models were often singular or failed to converge, and thus we report the random-intercept-only models here for consistency across the models. In order to maximize the variance accounted for by the model, all predictors were included in each model. Because all predictors were included simultaneously, regression coefficients reflect each predictor's unique association with network activity, accounting for the other predictors. Analyses followed the subsequent equation:

Activity∼narrativedetails+perceptualdetails+valence+narrative*valence+perceptual*valence+(1|subject).

This model was fit individually for each network, where network activity corresponded to the average across ROIs within a network. Due to the categorical nature of “valence” (positive, neutral, negative), analyses were first performed using “neutral” as the reference category and then rerun using “positive” as the reference category to capture any additional differences between positive and negative valence that could not be tested using “neutral” as the reference category. For each effect, *p* values were adjusted for multiple comparisons using the Benjamini–Hochberg method with a false discovery rate (FDR) of 0.05 (Benjamini and Hochberg, 1995), taking into account the tests for each network and MTL ROI. Values for narrative and perceptual details were mean centered within participants to better account for between-subject variability. In order to examine the effect of narrative and perceptual details beyond a simple difference between remembered and forgotten videos (no details), our mixed-effects analyses included only trials classified as “remembered” as indicated by participant rating of recall success at delayed retrieval. We also explored whether these results held up when considering all trials (remembered and forgotten). Note that all reported results remained significant except for one finding noted in the Results section.

### Representational similarity analysis

In order to assess the reactivation of event-specific information during recall, a representational similarity analysis (RSA; Kriegeskorte et al., 2008) was conducted to compare multivoxel pattern similarity between corresponding trials in the template and cued recall phases (Fig. 4*a*). The spatial pattern evoked by each recall trial was compared with the pattern for each template-phase trial using Pearson's correlation, and resulting correlation values were Fisher *z*-transformed. Event-specific reactivation was defined by the difference between same-video comparisons and different-video comparisons, computed separately for each recall trial. Same-video comparisons involved trial correlations that were matched on video, while different-video comparisons involved differing videos but were matched for valence and whether they were remembered or forgotten in the delayed recall task. Note that this was computed separately for each of the three recall trials associated with each video. This measure was evaluated separately for each parcel and then combined for the three default mode subnetworks. Trial-wise network values were entered into a regression model similar to the one used for the univariate analyses, with the *z*-transformed difference between same- and different-video trials included as the outcome variable. We also ran a model containing only the detail predictors to examine the effects without the complexity of valence reference category-related effects. Each effect was adjusted for multiple comparisons using the Benjamini–Hochberg method with a FDR of 0.05.

In order to capture the consistency, or stability, of event-specific memory representations across recalls, another RSA was conducted to examine multivoxel pattern similarity across the three recall rounds in the cued recall task (Fig. 5*a*). The spatial pattern of activity for each recall-phase trial was correlated with that of every other recall-phase trial using Pearson's correlation, and resulting correlation values were Fisher *z*-transformed. For each trial, same-video comparisons were made with patterns corresponding to the same video across the other rounds, and different-video comparisons were made with different videos from the other rounds that were matched for valence and whether they were remembered or forgotten in the delayed recall task. Recall stability was calculated as the difference between same-video comparisons and different-video comparisons, evaluated separately for each parcel and then combined for the three default mode subnetworks. Trial-wise values were saved out for each ROI using the same atlas and ROIs as in the univariate analysis described previously. As in the other RSA approach, we used a mixed-effects regression model mirroring our univariate analyses and then also examined a model which included only narrative and perceptual details. The *z*-transformed difference between same- and different-video trials was included as the outcome variable. This model was run for each of the three default mode subnetworks, and each effect was adjusted for multiple comparisons using the Benjamini–Hochberg method with a FDR of 0.05 (Benjamini and Hochberg, 1995). For both the template–recall and recall–recall approaches, one-sample *t* tests were computed to test whether event-specific reactivation (averaged across trials for each participant) in each of the DMN subnetworks was greater than zero.

## Results

### Valence effects on memory details

One day after encoding news videos and covertly retrieving them in the MRI, participants were asked to overtly recall as many details as possible about each video. To evaluate whether these memory measures at delayed recall related to in-scanner behavioral measures at cued recall, we related these details to participants' success ratings collected after each trial of cued recall in the MRI while accounting for the effects of emotional valence. Mean success ratings significantly predicted delayed vividness ratings (*β* = 0.720, *t*_(989)_ = 5.161, *p* < 0.001) as well as the number of perceptual and narrative details retained after 1 d (perceptual: *β* = 0.341, *t*_(989)_ = 2.163, *p* < 0.05; narrative: *β* = 0.439, *t*_(989)_ = 2.827, *p* < 0.01), indicating that Day 1 subjective memory success relates to the strength of memory on Day 2. Participants generally reported high levels of success among trials that were successfully recalled the following day (i.e., those included in the present analyses): 86.8% of trials received a rating of 4 or 5 on the 1–5 success scale (Extended Data Fig. 1-1). These findings indicate that participants were generally successful in bringing video details to mind during the scan and that their in-scanner recall was likely to correspond with the details recalled in the delayed test.

We then examined the effect of video valence (positive, neutral, negative) on the average number of overall, narrative, and perceptual details recalled by means of an ANOVA. There was a significant effect of valence on overall correct details remembered (*F*_(2,58)_ = 4.32, *p* < 0.05, $\eta_{G}^{2}=0.014$), indicating that participants recalled more correct details for positive videos (*M* = 7.95, SD = 3.95) than for neutral (*M* = 7.18, SD = 3.47, *t*_(29)_ = 3.028, *p* < 0.01), with no significant differences for negative (*M* = 6.20, SD = 4.71) compared with positive (*t*_(29)_ = 1.20, *p* = 0.24) or neutral (*t*_(29)_ = 1.73, *p* = 0.10).

We then distinguished between narrative and perceptual details to examine any distinct effects within detail types. Here, we found a significant effect of valence (*F*_(2,58)_ = 22.64, *p* < 0.001, $\eta_{G}^{2}=0.13$; Fig. 1*d*), such that more narrative details were recalled from positive videos (*M* = 4.89, SD = 2.21, *t*_(29)_ = 6.063, *p* < 0.001) and negative videos (*M* = 4.70, SD = 2.31, *t*_(29)_ = 5.404, *p* < 0.001) than neutral (*M* = 3.90, SD = 1.87). There were no differences between positive and negative videos (*t*_(29)_ = 1.03, *p* = 0.31). For perceptual details, on the other hand, there was a memory benefit for neutral videos (*F*_(2,58)_ = 3.87, *p* < 0.01, $\eta_{G}^{2}=0.009$; Fig. 1*d*), such that participants recalled more perceptual details for neutral videos (*M* = 2.43, SD = 2.68) compared with negative (*M* = 2.09, SD = 2.26, *t*_(29)_ = −2.79, *p* < 0.01) or positive videos (*M* = 2.08, SD = 2.32, *t*_(29)_ = 2.509, *p* < 0.05). There were no differences between positive and negative videos (*t*_(29)_ = 0.17, *p* = 0.86).

**Figure 1.**
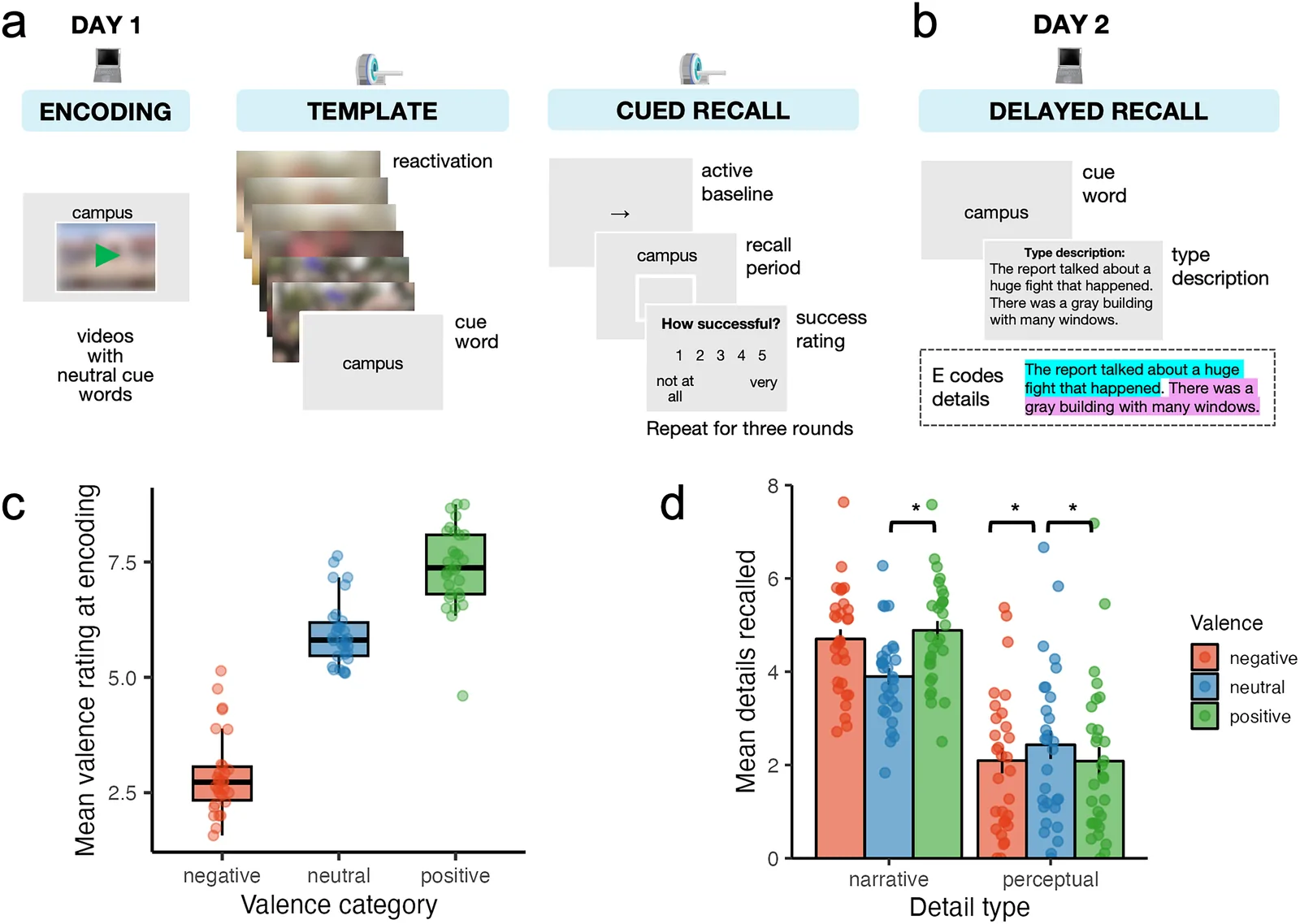
Experimental paradigm*.* ***a***, On experiment Day 1, participants completed encoding outside of the scanner, followed by template and cued recall in an MRI scanner. Video images have been blurred in this figure for copyright reasons. See Extended Data Figure 1-1 for characterization of in-scanner success ratings. ***b***, On Day 2, participants completed delayed recall outside of the scanner. ***c***, Subjective video valence ratings at encoding by valence category. Boxplots show the distribution of ratings for each valence category, with the boxplots showing the median and the interquartile range across participants and with individual data points representing each participant. ***d***, Average number of narrative and perceptual details recalled per video by valence category. More narrative details were remembered for positive than neutral events, while more perceptual details were remembered for neutral than positive or negative events. * indicates *p* < 0.05. Error bars indicate standard error of the mean. Points reflect average values for each participant.

10.1523/ENEURO.0146-26.2026.f1-1 Figure 1-1 Figure 1-1. Behavioral characterization and neural correlates of success ratings. **a)** Success rating distribution by emotional valence. To characterize participants’ subjective success during encoding, we examined the distribution of in-scanner success ratings. Participants generally reported high levels of success: among trials that were successfully recalled the following day (i.e., those included in the present analyses), 86.8% received a rating of 4 or 5 on the 1-5 success scale. We next tested whether perceived success differed by emotional valence using a linear mixed-effects model predicting success ratings from video valence. Success ratings varied significantly by valence: positive events were rated as more successful than neutral events (*β* = 0.129, *t*(2671.5) = 3.551, *p* < .001), neutral events were rated as more successful than negative events (*β* = -0.129, *t*(2671.8) = -3.486, *p* < .001), and positive events were rated as more successful than negative events (*β* = 0.259, *t*(2672) = 6.983, *p* < .001). Thus, while participants generally reported high success overall, their subjective perception of success varied by emotional valence. **b)** To investigate how default mode subnetwork activity was related to in-scanner success ratings, we ran a linear mixed-effects model predicting univariate activation from success, valence, and their interaction. The medial temporal subnetwork showed a significant positive association with success ratings (*β* = 0.055, *t*(2473.34) = 4.62, *p_FDR_* < .001), indicating that greater perceived success during in-scanner recall was associated with higher activity in the medial temporal subnetwork. No other DMN subnetwork showed significant effects of success or success-by-valence interactions. **c)** We conducted an analogous analysis predicting recall stability measures. Across DMN subnetworks, there was no main effect of success ratings on recall stability. However, an interaction was present in core midline and medial temporal subnetwork, such that higher success ratings for positive events were associated with lower recall stability (core midline: *β* = -0.013, *t*(2517.86) = -2.828, *p_FDR_* < .05; medial temporal: *β*= -.011, *t*(2516.69) = -2.540, *p_FDR_* < .05). When the same analysis was conducted using template-recall similarity as the outcome variable, we observed no significant relationships with subjective success. Download Figure 1-1, TIF file.

Thus, our results indicate differential effects of valence on memory for narrative versus perceptual details, in which narrative details were remembered best for positive and negative videos whereas perceptual details were recalled best for neutral videos. Importantly, narrative details and perceptual details were only moderately correlated with each other across participants (*r* = 0.376, *p* < 0.05), suggesting that there may be separate mechanisms by which narrative and perceptual details contribute to total remembering.

### Activity related to memory for narrative and perceptual details

In order to examine how default mode subnetwork activity relates to the contents of later memory and the valence of the remembered event, activity was first averaged across ROIs for each of the dorsomedial, medial temporal, and core midline subnetworks. Mixed-effects linear regression analyses were used to predict trial activity as a function of valence and the number of recalled narrative and perceptual details.

We found that activity in the dorsomedial subnetwork was sensitive to negative valence, such that negative videos produced higher activity in the dorsomedial network than neutral videos did (*β* = 0.065, CI: 0.03–0.1, *t*_(2724.064)_ = 3.678, *p*_FDR_ = 0.001; Fig. 2*a*,*c*). Furthermore, we found that core midline activity was significantly associated with positive valence compared with neutral (*β* = 0.041, CI: 0.01–0.07, *t*_(2722.820)_ = 2.964, *p*_FDR_ = 0.015). Note that this latter finding was specific to analyses limited to remembered trials and did not emerge when combining remember and forgotten trials together. Core midline and dorsomedial subnetwork activity was not related to memory for perceptual details, and no subnetwork showed a relationship with narrative details. In contrast, medial temporal subnetwork activity scaled with the number of perceptual details recalled (*β* = 0.011, CI: 0.00–0.02, *t*_(2726.857)_ = 2.509, *p*_FDR_ = 0.042; Fig. 2*d*), regardless of the emotional valence of the memory. All other effects across networks were nonsignificant, *p*_FDR_ > 0.05. When the reference category was switched to positive to assess positive–negative comparisons, no additional significant comparisons emerged (*p*_FDR_ > 0.05), and the relationship with perceptual details became nonsignificant, likely due to a numerically weaker relationship for the positive reference category. Providing further support for the relationship with perceptual details recalled, we also found that medial temporal subnetwork activity was positively associated with success ratings during in-scanner recall (Extended Data Fig. 1-1).

**Figure 2.**
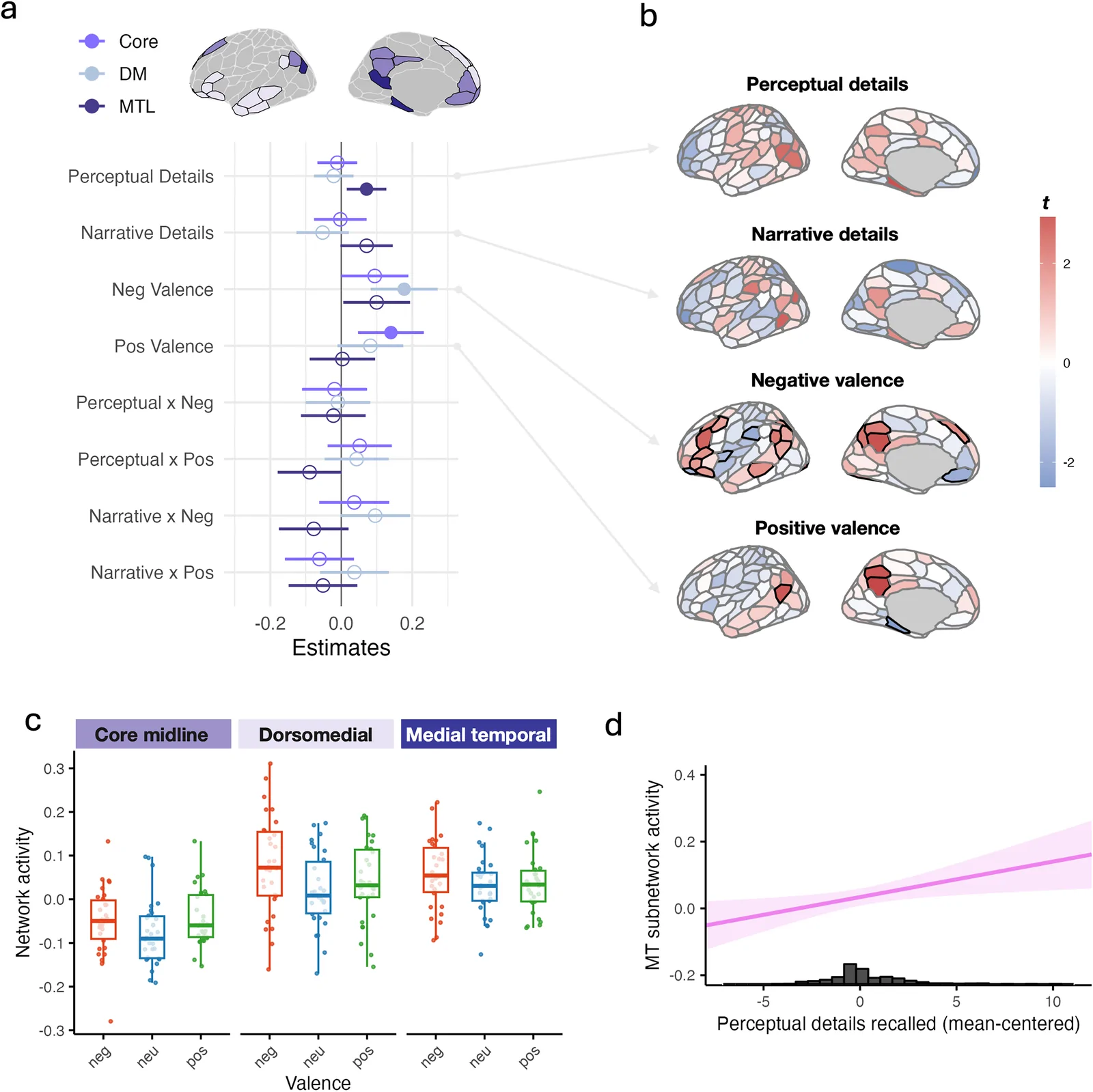
DMN activity related to remembered event features. ***a***, Mixed-effects model regression estimates for each DMN subnetwork with network activity as the outcome variable. Error bars indicate the 95% confidence interval for each estimate. Filled circles denote FDR-corrected *p* < 0.05. Networks are displayed using the Schaefer parcellation (Schaefer et al., 2018) with the Yeo 17-network labels (Yeo et al., 2011). See Extended Data Figure 2-1 for parcel-level correlations within and between networks. ***b***, Whole-brain mixed-effects model results for the four main predictors, displayed with the Schaefer parcellation (Schaefer et al., 2018). Parcels with significant *t* values after FDR correction are outlined in black. See Extended Data Figure 2-2 for visualization of parcel-level effects grouped by DMN subnetwork. ***c***, DMN subnetwork activity by valence. Boxplots represent the distribution of average network activity for each valence category, with the boxplots showing the median and the interquartile range across participants and with individual data points representing each participant. ***d***, Medial temporal subnetwork activity scales with the number of perceptual details recalled. Shading denotes the 95% confidence interval around the predicted values. The mixed-effects model regressor corresponding to this comparison was significant at FDR-corrected *p* < 0.05. Histogram represents the mean-centered distribution of perceptual details recalled, including trials from all participants.

10.1523/ENEURO.0146-26.2026.f2-1 Figure 2-1 Parcel-level correlations within and between networks. **a)** To assess whether network assignments captured meaningful co-variance among parcels in how they were engaged during recall, we computed pairwise correlations of task-related activity (across trials, using the trial averages from our primary analyses). We then tested whether the Fisher z transformed correlations were greater for within-subnetwork parcel pairs than between-subnetwork pairs by averaging within- and between- correlations for each subject and comparing them using a paired-samples t-test. We found that parcel pairs in the same subnetwork showed significantly higher average correlations than those across subnetworks, *t*(29) = 19.735, *p* < .001, indicating strong functional cohesion within each subnetwork during recall. **b)** Matrix displays the Fisher z-transformed Pearson’s *r* correlation between each parcel defined in the DMN. Black lines indicate separation between the three networks. Darker colors indicate a stronger positive correlation. Download Figure 2-1, TIF file.

10.1523/ENEURO.0146-26.2026.f2-2 Figure 2-2 Activity related to remembered event features: Parcel-level effects. Plots show t-values from the linear mixed-effects model predicting activity for each individual parcel, with parcels grouped by default mode subnetwork and labeled using Schaefer atlas abbreviations (Schaefer et al., 2018). The corresponding predictor is labeled at the top of each plot. Filled circles indicate parcel-level significance at FDR corrected *p* < .05. We found that valence-related effects were distributed but most prevalent within the core and dorsomedial subnetworks, consistent with our network-level findings. No individual DMN parcel showed significant sensitivity to narrative or perceptual detail. However, visual inspection of parcel-level effects suggests that the medial temporal subnetwork shows coherent recruitment specifically for detail-related effects, suggesting possible subnetwork-level specialization. Download Figure 2-2, TIF file.

To characterize these effects further and to examine the involvement of individual parcels in our network-level effects, we ran the same models for all 200 parcels in the brain. Here, we focus on the results for DMN parcels (Table 1); results for non-DMN parcels are shown in Figure 2*b* and Extended Data Table 1-1. We found that valence-related effects were distributed but most prevalent within the core and dorsomedial subnetworks (Table 1, Extended Data Fig. 2-2), consistent with our network-level findings. No individual DMN parcel showed significant sensitivity to narrative or perceptual detail. However, visual inspection of parcel-level effects suggests that the medial temporal subnetwork shows coherent recruitment specifically for detail-related effects, suggesting that subnetwork-level specialization may emerge from moderate activity changes distributed across the network rather than isolated parcel effects (Extended Data Fig. 2-2).

10.1523/ENEURO.0146-26.2026.t1-1 Table 1-1 Significant associations between parcel activity and predictors across the whole brain, using a threshold of FDR-corrected *p* < .05. Here, DefaultA corresponds to core midline, DefaultB to dorsomedial subnetwork, and DefaultC to medial temporal subnetwork. Network and region labels are from the Schaefer atlas. LH = left hemisphere, RH = right hemisphere. Download Table 1-1, DOCX file.

**Table 1.** Significant parcel-level outcomes across all parcels in the DMN for the main mixed-effects model estimates, where FDR-corrected *p* < 0.05

Term	Network	Region label	Hemi	Estimate	df	*t*	*p* (FDR)
Negative valence	Core midline	IPL_1	LH	0.1040	2,720	2.65	*p* < 0.05
	Core midline	IPL_1	RH	0.1160	2,720	4.54	*p* < 0.001
	Core midline	PFCm_1	LH	−0.0394	2,720	−3.23	*p* < 0.01
	Core midline	pCunPCC_1	LH	0.0735	2,720	5.11	*p* < 0.001
	Core midline	pCunPCC_1	RH	0.0878	2,720	5.77	*p* < 0.001
	Core midline	pCunPCC_3	LH	0.0573	2,720	3.82	*p* < 0.01
	Dorsomedial	AntTemp_1	RH	0.0608	2,720	4.48	*p* < 0.001
	Dorsomedial	IPL_1	LH	0.1100	2,720	3.99	*p* < 0.001
	Dorsomedial	PFCd_1	RH	0.1280	2,720	4.45	*p* < 0.001
	Dorsomedial	PFCd_3	LH	0.0714	2,720	3.44	*p* < 0.01
	Dorsomedial	PFCv_1	LH	0.0499	2,720	2.99	*p* < 0.05
	Dorsomedial	PFCv_1	RH	0.1100	2,720	4.70	*p* < 0.001
	Dorsomedial	PFCv_2	LH	−0.0504	2,720	−2.93	*p* < 0.05
	Dorsomedial	PFCv_3	LH	0.0978	2,720	2.91	*p* < 0.05
	Dorsomedial	PFCv_4	LH	0.0773	2,720	3.17	*p* < 0.05
	Dorsomedial	Temp_1	RH	0.1600	2,720	6.10	*p* < 0.001
	Dorsomedial	Temp_4	LH	0.0871	2,720	3.29	*p* < 0.01
	Medial temporal	IPL_1	RH	0.0677	2,720	2.63	*p* < 0.05
Positive valence	Core midline	IPL_1	RH	0.0795	2,720	3.17	*p* < 0.05
	Core midline	pCunPCC_1	LH	0.0684	2,720	4.85	*p* < 0.001
	Core midline	pCunPCC_1	RH	0.0858	2,720	5.75	*p* < 0.001
	Core midline	pCunPCC_3	LH	0.0664	2,720	4.52	*p* < 0.001
	Dorsomedial	AntTemp_1	RH	0.0531	2,720	3.99	*p* < 0.01
	Dorsomedial	PFCd_1	RH	0.0983	2,720	3.48	*p* < 0.05
	Dorsomedial	Temp_1	RH	0.0931	2,720	3.62	*p* < 0.01
	Medial temporal	PHC_1	LH	−0.0245	2,720	−3.48	*p* < 0.05

Region labels are from the Schaefer atlas. LH, left hemisphere; RH, right hemisphere. See Extended Data Table 1-1 for significant parcel-level outcomes across the whole brain.

Similar analyses were conducted for the hippocampal and amygdala ROIs, motivated by the known role of the hippocampus and amygdala in emotional memory (Phelps, 2004) and potential dissociations among hippocampal subregions in emotional memory (Ritchey et al., 2019). Overall hippocampal activity was significantly associated with the number of remembered perceptual details (*β* = 0.005, CI: 0.00–0.01, *t*_(2728.025)_ = 2.192, *p*_FDR_ = 0.047). A hippocampal subregion analysis revealed that this relationship was significant for the left anterior hippocampus (*β* = 0.008, CI: 0.00–0.01, *t*_(2722.682)_ = 2.544, *p*_FDR_ = 0.044; Fig. 3) but not for the hippocampal body or tail. Amygdala activity also significantly scaled with the number of remembered perceptual details (*β* = 0.007, CI: 0.00–0.01, *t*_(2726.404)_ = 2.389, *p*_FDR_ = 0.042; Fig. 3), and in the models directly comparing positive and negative valence, amygdala activity was significantly associated with positive valence compared with negative (*β* = −0.028, CI: −0.05–−0.01, *t*_(2721.456)_ = −2.968, *p*_FDR_ = 0.003). Neither hippocampal nor amygdala activity was related to the number of narrative details recalled. All other effects across models and ROIs were nonsignificant (all *p*_FDR_ > 0.05).

**Figure 3.**
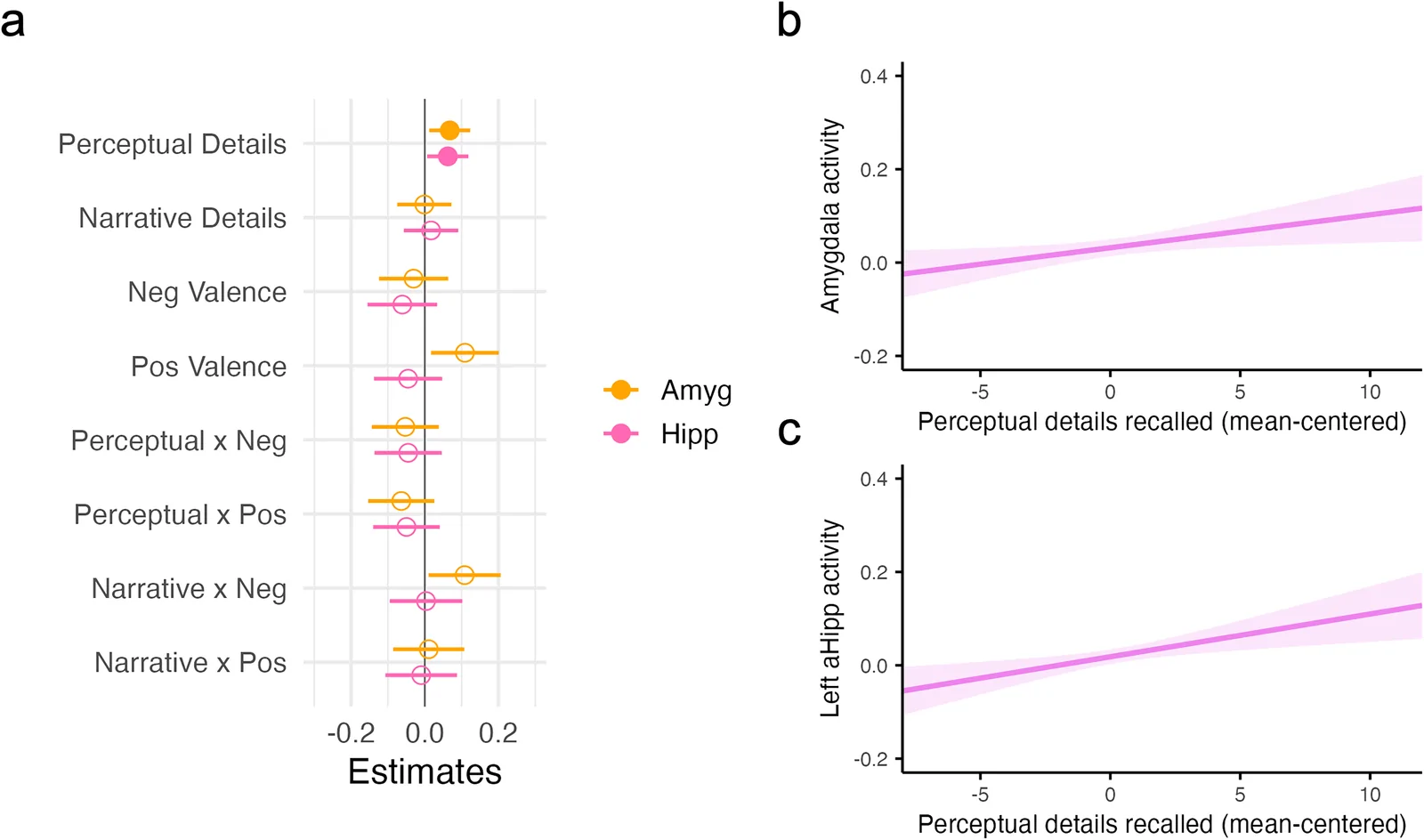
Amygdala and hippocampal activity related to remembered event features. ***a***, Mixed-effects model estimates for the amygdala and hippocampus with activity as the outcome variable. Error bars indicate the 95% confidence interval for each estimate. Filled circles denote FDR-corrected *p* < 0.05. ***b***, Amygdala and (***c***) left anterior hippocampus activity scale with the amount of perceptual details recalled. Shading denotes the 95% confidence interval around the predicted values. The mixed-effects model regressor corresponding to this comparison was significant at FDR-corrected *p* < 0.05.

Together, these results suggest that the dorsomedial and core midline subnetworks are especially responsive to memories for emotional events, whereas the medial temporal subnetwork, hippocampus, and amygdala are more active when recalling memories with perceptual details that persist in memory.

### Event-specific patterns of activation and their relation to memory quality

To determine how the strength of event-specific patterns in the DMN is related to recapitulation of different types of details, we conducted a RSA assessing multivoxel pattern similarity between the template and cued recall phases of the experiment and its relationship to detail type and emotional valence. We reasoned that this measure of template–recall similarity would be sensitive to the reactivation of high-fidelity, event-specific details from encoding that span the template and recall phases. Across conditions and in each of the DMN subnetworks, there was evidence for event-specific reactivation (Fig. 4*b*), such that same-video similarity was greater than different-video similarity (core midline: *t*_(29)_ = 3.94; dorsomedial: *t*_(29)_ = 3.52; medial temporal: *t*_(29)_ = 5.13; all *p* < 0.001). However, this effect was not significantly associated with the number of narrative or perceptual details or with negative or positive valence compared against neutral (*p*_FDR_ > 0.05). When comparing negative and positive valence directly (i.e., with positive valence as the reference), there was an interaction between valence and narrative detail, such that reactivation of medial temporal subnetwork areas was more strongly associated with narrative detail for negative videos compared with positive (*β* = 0.005, CI: 0.00–0.01, *t*_(2788.307)_ = 3.03, *p*_FDR_ = 0.007; Fig. 4*c*). Finally, we note that there was a significant relationship with perceptual details in the positive reference model that was not observed in the neutral reference model, likely due to numerical differences in the strength of the relationship for the different valence categories. Thus, to clarify these effects, we additionally ran a simpler model collapsing across valence, including fixed effects only for narrative and perceptual details, and found that there was no significant relationship between reactivation in DMN areas and memory for either kind of detail (*p*_FDR_ > 0.05). These findings suggest that reactivation in the medial temporal network may be more influenced by narrative detail for negative compared with positive events but that, otherwise, there was little association between template–recall similarity and memory quality.

**Figure 4.**
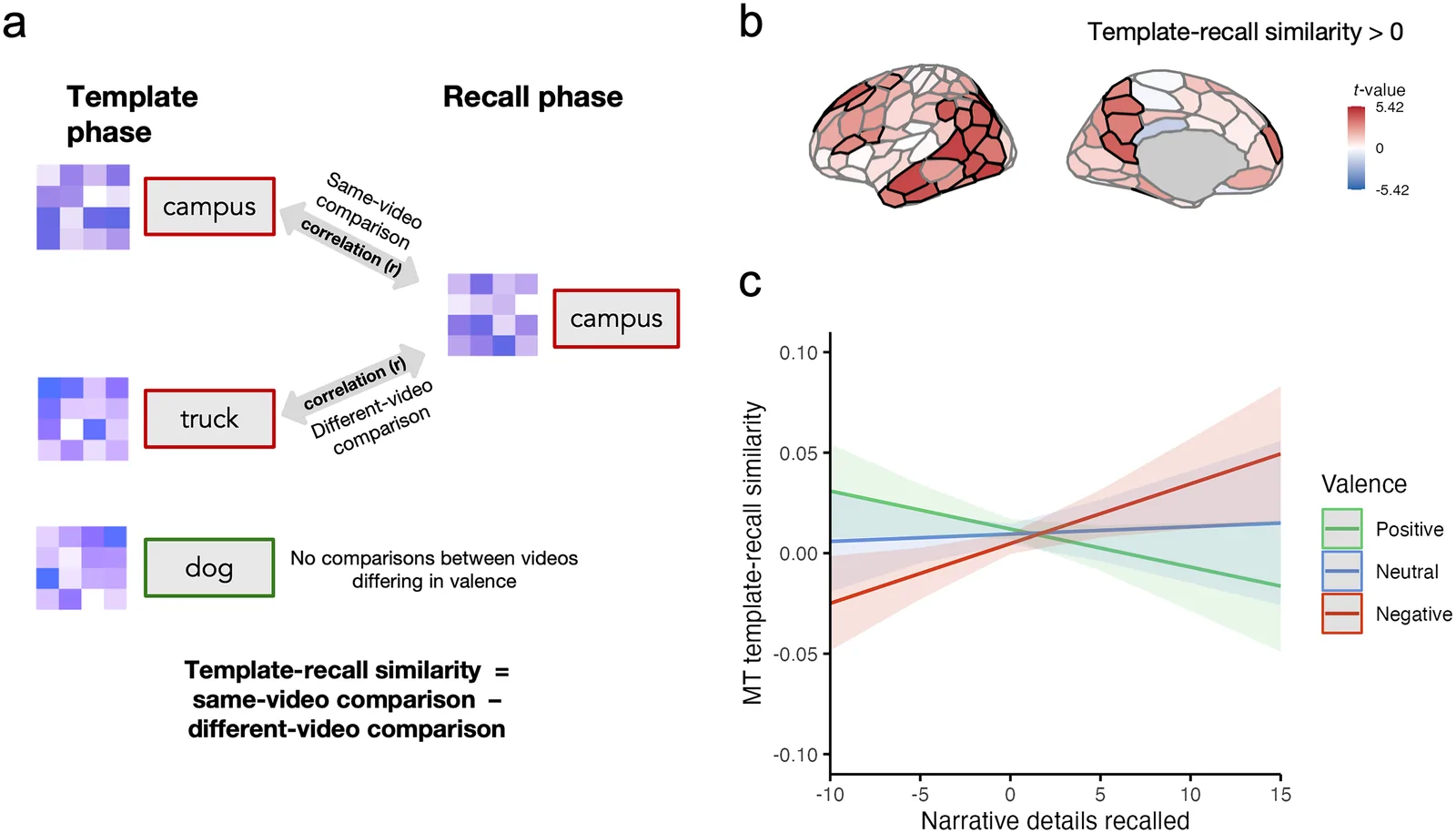
Template–recall similarity. ***a***, Template–recall similarity RSA method. Same-video comparisons were calculated by correlating activity patterns elicited by the same video during the template and recall phases. Different-video comparisons were calculated by correlating each trial's recall activity pattern with the template activity pattern of other videos matched on valence. ***b***, Whole-brain results of a one-sample *t* test of template–recall similarity (same-video similarity minus different-video similarity) displayed with the Schaefer parcellation (Schaefer et al., 2018). Parcels with significant *t* values after FDR correction are outlined in black. See Extended Data Figure 4-1 for visualization of parcel-level template–recall similarity effects grouped by DMN subnetwork. ***c***, Template–recall similarity in the medial temporal subnetwork was more strongly positively related to narrative details for negative compared with positive valenced events. Shading denotes the 95% confidence interval around the predicted values. The mixed-effects model interaction term corresponding to this comparison was significant at FDR-corrected *p* < 0.05.

10.1523/ENEURO.0146-26.2026.f4-1 Figure 4-1 Template-recall similarity: Parcel-level effects. Plots show t-values from the linear mixed-effects model predicting template-recall similarity for each individual parcel, with parcels grouped by default mode subnetwork and labeled using Schaefer atlas abbreviations (Schaefer et al., 2018). The corresponding predictor is labeled at the top of each plot. Filled circles indicate significance at FDR corrected *p* < .05. Only one parcel showed evidence of being modulated by the features of the remembered videos: the right parahippocampal cortex showed reactivation more for positive than neutral videos, with a similar non-significant pattern for negative videos. Download Figure 4-1, TIF file.

Because event patterns may be transformed from perception to recall (Xiao et al., 2017; Favila et al., 2020; Xue, 2022), we additionally assessed how event-specific memory representations were maintained across the three retrieval rounds in the cued recall phase. We reasoned that this measure of recall–recall similarity would indicate the stability of the event representation over repeated retrievals. Across conditions and in each of the DMN subnetworks, there was evidence for event-specific recall stability (Fig. 5*b*), such that same-video similarity was greater than different-video similarity (core midline: *t*_(29)_ = 5.13; dorsomedial: *t*_(29)_ = 6.09; medial temporal: *t*_(29)_ = 4.60; *p* < 0.002). In addition, recall stability was significantly modulated by the contents of memory. Recalling positively valenced events was associated with more stable recall patterns in core midline and dorsomedial subnetworks than recalling neutral events (core midline: *β* = 0.009, CI: 0.00–02, *t*_(2763.135)_ = 2.813, *p*_FDR_ = 0.009; dorsomedial: *β* = 0.012, CI: 0.00–0.02, *t*_(2765.065)_ = 3.213, *p*_FDR_ = 0.004; Fig. 5*c*). Recall stability was also stronger in core midline for negative event recall compared with neutral (*β* = 0.009, CI: 0.00–02, *t*_(2764.231)_ = 2.745, *p*_FDR_ = 0.028, Fig. 5*c*). While there were no relationships with narrative or perceptual detail in the full model, a model containing only narrative and perceptual details as predictors showed that perceptual detail recall scaled with recall stability in the medial temporal network (*β* = 0.002, CI: 0.00–0.00, *t*_(2763.168)_ = 3.34, *p*_FDR_ = 0.003). All other effects across models and networks were nonsignificant, all *p*_FDR_ > 0.05. These results suggest that event-specific representations of valence memory are maintained across retrievals in the dorsomedial and core midline subnetworks, whereas representations in the medial temporal network are sensitive to perceptual details independently of valence.

**Figure 5.**
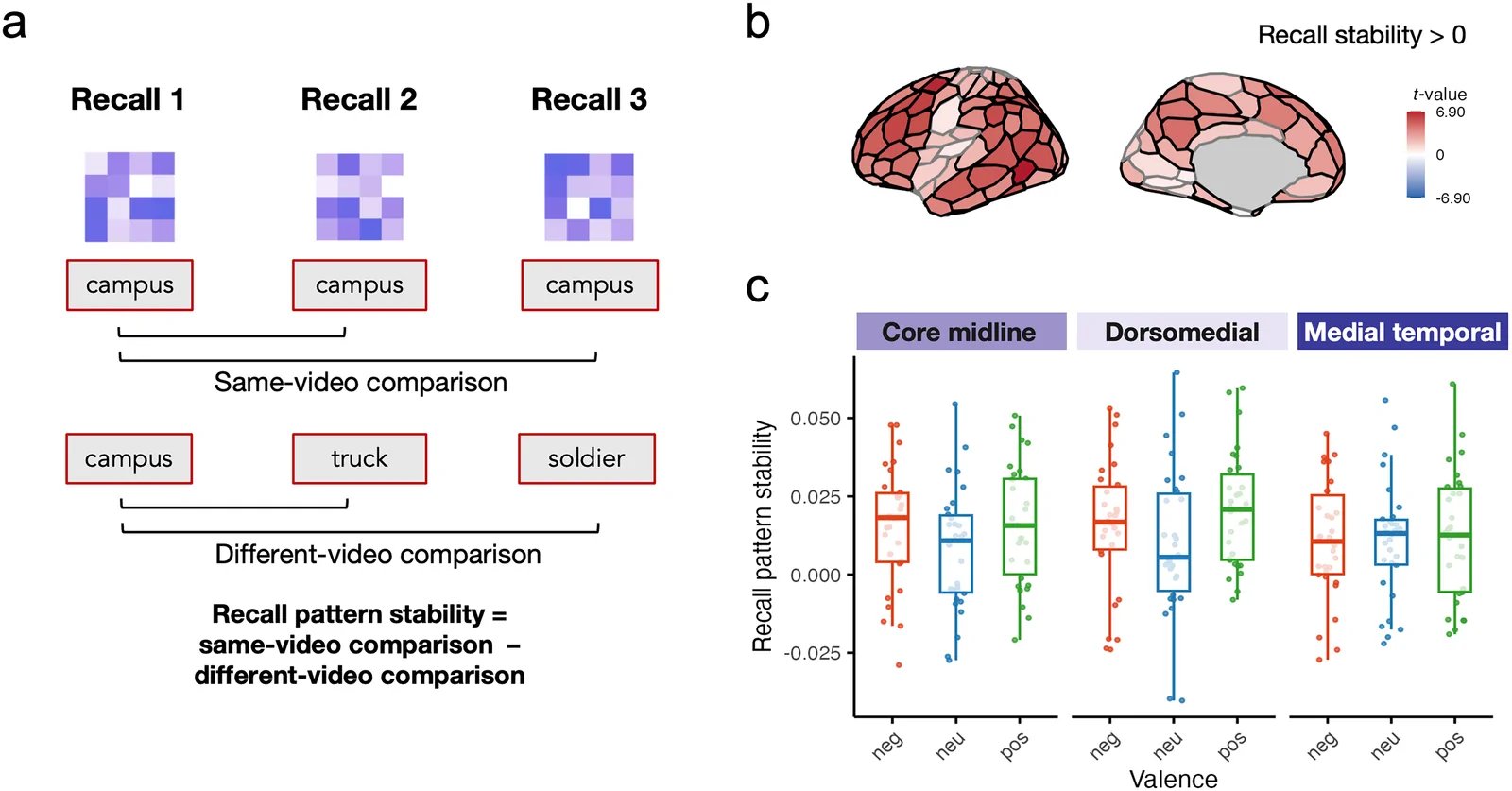
Recall stability. ***a***, Recall stability RSA method. Each trial's retrieval pattern was compared with other repetitions of the same video recall (i.e., in different rounds) to obtain the average same-video comparison. The trial's correlations with other valence-matched, different-video recall trials were averaged to obtain the different-video comparison. ***b***, Whole-brain results of a one-sample *t* test of recall pattern stability (same-video comparisons minus different-video comparisons) displayed with the Schaefer parcellation (Schaefer et al., 2018). Parcels with significant *t* values after FDR correction are outlined in black. See Extended Data Figure 5-1 for visualization of parcel-level recall stability effects grouped by DMN subnetwork. ***c***, DMN subnetwork recall stability by valence. Boxplots represent the distribution of average network recall stability for each valence category, with the boxplots showing the median and the interquartile range across participants and with individual data points representing each participant.

10.1523/ENEURO.0146-26.2026.f5-1 Figure 5-1 Recall stability: Parcel-level effects. Plots show t-values from the linear mixed-effects model predicting recall stability for each individual parcel, with parcels grouped by default mode subnetwork and labeled using Schaefer atlas abbreviations (Schaefer et al., 2018). The corresponding predictor is labeled at the top of each plot. Filled circles indicate significance at FDR corrected *p* < .05. We found that parcels in the dorsomedial and core networks showed greater recall stability for positive memories compared to neutral memories, with a similar trend for negative compared to neutral memories. One region in the dorsomedial network, the ventromedial prefrontal cortex, was also sensitive to perceptual details recalled. Download Figure 5-1, TIF file.

To examine the contribution of individual parcels to these results, we re-ran both our RSA approaches across all 200 parcels in the brain. We found that parcels in the dorsomedial and core networks showed greater recall stability for positive memories compared with neutral memories, with a similar trend for negative compared with neutral memories (Extended Data Fig. 5-1). One region in the dorsomedial network, the ventromedial prefrontal cortex, was also sensitive to perceptual details recalled. For template–recall similarity, only one DMN parcel showed evidence of being modulated by the features of the remembered videos (Extended Data Fig. 4-1): the right parahippocampal cortex showed greater template–recall similarity for positive than neutral videos, with a similar nonsignificant pattern for negative compared with neutral videos (Table 2). Whole-brain results outside of the DMN can be found in Extended Data Table 2-1. These results indicate that recall stability in parcels across the dorsomedial and core networks is generally modulated by valence but that the relationships with details may be more variable across parcels.

10.1523/ENEURO.0146-26.2026.t2-1 Table 2-1 Significant associations between our two RSA measures and predictors across the whole brain, using a threshold of FDR-corrected *p* < .05. Here, DefaultA corresponds to core midline, DefaultB to dorsomedial subnetwork, and DefaultC to medial temporal subnetwork. Network and region labels are from the Schaefer atlas. LH = left hemisphere, RH = right hemisphere. Download Table 2-1, DOCX file.

**Table 2.** Significant parcel-level outcomes across all parcels in the DMN for the mixed-effects model estimates from each RSA approach, where FDR-corrected *p* < 0.05

Term	Network	Region label	Hemi	Estimate	df	*t*	*p* (FDR)
Parcel-level template–recall reactivation							
Positive valence	Medial temporal	PHC_1	RH	0.0114	2,780	4.06	*p* < 0.05
Parcel-level recall stability							
Perceptual details	Dorsomedial	PFCv_2	LH	0.00658	2,770	4.70	*p* < 0.001
Positive valence	Core midline	PFCd_1	RH	0.01610	2,770	3.29	*p* < 0.05
	Core midline	PFCm_2	LH	0.01930	2,770	2.95	*p* < 0.05
	Dorsomedial	PFCd_1	RH	0.03370	2,770	4.70	*p* < 0.001
	Dorsomedial	PFCd_3	LH	0.02210	2,770	3.95	*p* < 0.01
	Dorsomedial	PFCv_2	LH	0.02230	2,770	5.05	*p* < 0.001

Region labels are from the Schaefer atlas. LH, left hemisphere; RH, right hemisphere. See Extended Data Table 2-1 for significant parcel-level RSA outcomes across the whole brain.

## Discussion

The present study examined whether DMN contributions to recall vary by the valence and detailedness of memories. We found that the engagement of DMN subnetworks varied depending on the features persisting in memory: dorsomedial subnetwork activity was associated with memory for event valence, while medial temporal subnetwork activity scaled with the number of perceptual details recalled. Amygdala and anterior hippocampus activity also scaled with remembered perceptual details, with the amygdala also showing greater activity for positive versus negative events. In addition to these activation differences, event-specific patterns in the DMN and, in particular, their stability across recall repetitions were also affected by memory valence and detail. These findings demonstrate that DMN contributions to recall are modulated by emotional valence and the persistence of specific details in memory.

Our finding that medial temporal subnetwork activity was related to the perceptual detailedness of memory aligns with previous studies implicating these regions in episodic retrieval. The medial temporal subsystem is involved in episodic memory and scene construction (Andrews-Hanna et al., 2010) and is more strongly connected to visual network regions than other DMN components (Barnett et al., 2021). Here, the relationship with perceptual details emerged at the network level: each parcel within the medial temporal subnetwork showed a similar profile of results, suggesting that the network-level effect was not carried by any individual region but rather the network as a whole. Recall stability in the medial temporal subnetwork was also related to the amount of remembered perceptual details, suggesting that event-specific patterns in these areas are intertwined with perceptual aspects of memory. Contrary to our hypothesis, this relationship was not modulated by negative emotional valence, despite prior findings that negative valence biases visual processing (Kark and Kensinger, 2019) and enhances sensory recapitulation (Bowen et al., 2018). Behaviorally, neutral events were remembered with more perceptual detail than emotional events. This discrepancy may stem from the nature of our naturalistic news stimuli, where the central details were largely narrative aspects of the event, rather than vividly perceptual in nature (as compared with studies using threatening or graphic images). If emotion was primarily conveyed through narrative, rather than perceptual details, this may also explain the finding that valence modulated the relationship between narrative detail and template–recall similarity in the medial temporal subnetwork, with a stronger relationship for negative compared with positive stimuli. This unexpected finding suggests that high-fidelity memory reinstatement in these areas may be involved in prioritizing or maintaining narrative details for naturalistic negative events. This pattern of results also points to the importance of considering which kinds of details carry the emotional impact of the event.

The relationship between perceptual detail and anterior hippocampal recruitment is consistent with studies linking anterior hippocampus to greater pattern specificity (Tompary and Davachi, 2017; Ezzyat et al., 2018), scene construction (Zeidman and Maguire, 2016), and detailed episodic processes (Addis et al., 2011; Dandolo and Schwabe, 2018). Posterior hippocampus activity was unrelated to any of our predictors, and narrative detail did not relate to anterior or posterior hippocampus activity. Thus, in contrast with some prior proposals (Poppenk et al., 2013; Robin and Moscovitch, 2017; Sheldon et al., 2019), we found no evidence for differential sensitivity to narrative versus perceptual recall along the hippocampal long axis.

Our results support the hypothesis that the dorsomedial network represents the emotional valence of memory. Dorsomedial DMN subdivisions have been linked to emotional experience (Andrews-Hanna et al., 2010; Barnett et al., 2021), with the dmPFC as a key node (Kensinger and Ford, 2021). Because memories were recalled in response to neutral cue words, our findings reflect valence information arising from memory rather than external cues. The dorsomedial subnetwork appeared to be sensitive to emotional information in general rather than being valence-specific, as there were no significant differences in activity or recall stability when directly comparing positive and negative valence. Contrary to our original hypothesis, the dorsomedial subnetwork did not show a stronger association with narrative details for emotional memories. This, along with its involvement in emotional recall regardless of the number of remembered details, suggests that the dorsomedial subnetwork primarily provides an affective scaffold for memory, integrating emotional tone without directly calling upon specific narrative or perceptual components.

The core midline subnetwork showed a similar profile of activity to the dorsomedial subnetwork but with some differences related to emotional valence. Specifically, it was more active for positive than neutral events and exhibited more stable event-specific retrieval patterns for both negative and positive events compared with neutral. These results suggest a role in integrating affective experience into memory, consistent with evidence that core midline regions activate together during self-relevant affective decisions (Andrews-Hanna et al., 2010). These results point to similarities in dorsomedial and core midline contributions to emotional memory recall, consistent with recent proposals that these are not entirely distinct networks. Individual-level functional connectivity analyses have demonstrated that the core midline's prominence in group-averaged data results from anatomical interdigitation between the two primary networks identifiable at the individual level (Braga et al., 2019), and studies remain agnostic about a clear function for this region (Wen et al., 2020). Thus, the core midline's sensitivity to affective valence in this study may result from signal intermixing from the dorsomedial subnetwork.

Our findings contribute to existing evidence of functionally distinct DMN subnetworks supporting episodic memory and cognition (Andrews-Hanna et al., 2010; Ranganath and Ritchey, 2012; Barnett et al., 2021). Prior research suggests that dorsal DMN supports abstract representations while ventral DMN supports perceptually specific information (Spunt et al., 2016; Sheldon et al., 2019; Ritchey and Cooper, 2020; Andrews-Hanna and Grilli, 2021). Furthermore, dorsal and ventral DMN are modulated by the emotional valence and vividness, respectively, of imagined future events (Lee et al., 2021) and are differentially recruited when generating emotional versus semantic contextual associations (Souter et al., 2024). Here, we find that such differences extend to contents of episodic memory: whereas perceptual details may contribute to the episodic specificity of memory, valence may be tied to more abstract (i.e., less detailed) event representations. This pattern complements existing research showing that DMN engagement varies depending on the type of information being recalled, such as objects, characters, or places (Gilmore et al., 2021; Procida et al., 2025; Ritchey, 2026).

Surprisingly, DMN activity did not relate to the amount of narrative detail recalled, diverging from previous findings implicating lateral temporal and medial frontal regions in narrative retrieval (Binder et al., 2009; Baetens et al., 2014; Gurguryan and Sheldon, 2019). Although we had hypothesized that narrative detail recall would correspond to dorsomedial and core midline activation, there are a number of reasons why the current paradigm may have produced different results. It is possible that the types of narrative details supported by the dorsomedial subnetwork were not captured in participants' written recalls, as our measure focused on specific story details rather than general conceptual or schematic information, which might rely more strongly on frontal and temporal areas. Another possibility is that recall performance was influenced by factors beyond initial memory retrieval, such as forgetting over the 1 d delay between scanning and written recall. We found that in-scanner memory ratings strongly related to the number of details recalled the next day, suggesting a meaningful relationship between brain activity and the enduring contents of memory. However, there may have been additional details recalled during the scan that were forgotten during delayed recall, which would not be captured by our analysis approach. For example, consolidation processes occurring between the two tests could have differentially impacted certain types of details, for instance, weakening perceptual details while strengthening semantic gist (Robin and Moscovitch, 2017; Lifanov et al., 2021). Future research incorporating immediate tests and separate measures for general and specific memory content will help clarify how brain activity relates to the persistence of memory details across time.

Past work has identified event-specific patterns of activity in DMN regions, including the medial parietal cortex (Bird et al., 2015) and lateral parietal cortex (Kuhl and Chun, 2014), but less is known about how the maintenance of these patterns is influenced by memory contents. We used two approaches to characterize these patterns: one measuring recall pattern similarity to a high-fidelity video template and another examining recall pattern stability across repeated retrievals. Emotional valence enhanced recall stability in the dorsomedial subnetwork, while perceptual detail enhanced stability in the medial temporal subnetwork. Comparisons across recalls may capture transformations in the memory representation that distinguish it from perceptual experience (Favila et al., 2020; Xue, 2022). Our recall stability analysis further reveals what event-specific information is maintained consistently across three retrieval rounds. Repeated retrieval can promote a more stable memory trace over time (Antony et al., 2017; Ferreira et al., 2019), accelerating immediate consolidation processes while maintaining the availability of episodic information. Thus, the relationship between recall stability and memory features may reflect early consolidation processes that protect these memory details over time. While this measure informs the consistency of the representation across repeated retrievals, we did not directly examine the degree of representational change across the individual recall rounds. The current study was limited in answering this question because there were relatively few trials per round and because the effects of repeated retrieval were confounded with the effects of time. Future studies incorporating more trials could resolve these issues to determine whether different memory features are differentially transformed with repeated retrieval.

In contrast, template–recall similarity showed limited association with memory characteristics. It should be acknowledged that template-phase patterns should not be considered as a straightforward measure of encoding-related activity but rather a high-fidelity representation of what was previously encoded, incorporating both perceived and remembered information. Thus, template–recall similarity reflects how similar the recall patterns were to an event-specific instance of highly detailed retrieval. The current findings suggest that recall activity patterns may better capture memory quality than the template phase, where other nonremembered perceptual features may influence patterns more strongly, making template–recall similarity less sensitive to memory details that are preserved over time.

In conclusion, we identified functional differentiation among DMN subnetworks in supporting memories that persist over time. The dorsomedial subnetwork contributes to an emotional framework for event memory, whereas the medial temporal network is recruited during recall of perceptually detailed memories. By leveraging analyses of both activity and event-specific patterns, the results offer novel evidence for DMN heterogeneity in retrieving the emotional context and perceptual details of past events, informing theoretical accounts of how DMN networks work in tandem to reconstruct complex memory experiences.

## Synthesis

Reviewing Editor: Maria Rajah, Toronto Metropolitan University Decisions are customarily a result of the Reviewing Editor and the peer reviewers coming together and discussing their recommendations until a consensus is reached. When revisions are invited, a fact-based synthesis statement explaining their decision and outlining what is needed to prepare a revision will be listed below. The following reviewer(s) agreed to reveal their identity: Carlo Sestieri. Note: If this manuscript was transferred from JNeurosci and a decision was made to accept the manuscript without peer review, a synthesis may not be available.

Dear Author,

Thank you for submitting your manuscript "Remembered event features shape default mode network engagement during emotional memory recall" (eN-TNWR-0146-26X) to eNeuro.

I have read the manuscript and the reviewer comments. Reviewer 1 recommended 'Accept as is' and Reviewer 2 recommended Revise and Resubmit and had several requests for clarification on behavioral and had theoretical questions. Their full review is provided below.

I agree with Reviewer 2 that greater methodological clarity and and improved operational definition would benefit this manuscript (R2, Comments 1 - 4). I would request these comments be directly addressed in your revision. However, I do not think you need to change the theoretical framing of the study despite results not aligning with the framing of the current paper and its result to a different framework. As such, there is not need to directly address comments 7 and 8, but think you need to provide caveats related to R2 comments 5 and 6 in the discussion/limitations sections.

Based on these reviews, the recommendeation is Revise, and Reviewing Editor Review Only.

Kind regards,

Reviewing Editor

Dr. M. N. Rajah

Reviewer 2 Feedback:

Summary

This manuscript addresses a well-motivated question: how functionally distinct subnetworks of the default mode network (DMN) contribute to the retrieval of different features of emotional memories. Using a naturalistic paradigm in which participants encoded and later recalled emotionally valenced news videos, the authors combine univariate activation analyses with representational similarity analyses (RSA) to dissociate the contributions of the dorsomedial, medial temporal, and core midline subnetworks. The distinction between narrative and perceptual memory detail is a meaningful conceptual advance, and the use of both template-recall and recall-recall RSA approaches is commendable. However, several methodological and interpretive concerns require substantive attention before the manuscript is suitable for publication.

Major Concerns

1. Scoring protocol and inter-rater reliability.

The manuscript attributes the scoring protocol to Levine et al. (2002), but the category labels used here do not map directly onto the original Autobiographical Interview (AI) categories. If the protocol represents a modified version of the AI, the authors should state this explicitly and specify which categories were adapted, renamed, or newly introduced relative to the original. More critically, the manuscript provides no inter-rater reliability statistics anywhere, even though the central neuroimaging findings depend on the validity of these hand-scored behavioral measures. The authors should report intraclass correlation coefficients (ICCs) for all detail categories fed into the analyses (event, visual, and total correct) and describe how scoring disagreements were resolved. Finally, the manuscript states that several AI categories were excluded from analyses due to insufficient detail counts, but no descriptive statistics are provided for these excluded categories. Mean detail counts (with standard deviations) across videos and participants should be reported for all scored categories, both to document the scoring burden and to justify the exclusions.

2. Operationalization of emotional valence.

Emotional valence was entered into the neuroimaging models as a categorical variable derived from the normative stimulus classifications in Samide et al. (2020), rather than from participants' own trial-wise ratings collected at both encoding and delayed recall. Given that affective responses to naturalistic stimuli vary substantially across individuals, this choice conflates stimulus category with subjective emotional experience, potentially attenuating the association between neural activity and the emotional tone of the memory as actually experienced. The authors should: (i) justify the preference for normative categorical valence over continuous subjective ratings as the primary neuroimaging predictor; (ii) report the correspondence between normative category assignments and individual participants' valence ratings; and (iii) examine whether substituting or supplementing the categorical predictor with trial-wise subjective ratings changes the pattern of results.

3. Handling of forgotten and reappraisal trials in the GLM.

The manuscript states that analyses were restricted to remembered trials and that the 12 negative reappraisal trials were excluded from the present analyses. However, it is not stated whether forgotten trials and reappraisal trials were explicitly modeled as regressors of no interest in the first-level GLM. If they were simply omitted, their associated neural signal would remain in the residual time series and could contaminate the activity estimates for trials of interest. The authors should clarify how these trial types were handled at the GLM level and confirm whether their exclusion from the behavioral analyses was matched by appropriate nuisance modeling in the neuroimaging pipeline.

4. Causal direction and predictor independence in the neuroimaging models.

The regression models treat the number of narrative and perceptual details recalled one day after scanning as predictors of BOLD activity acquired the previous day. This inverts the temporal and causal relationship: memory detail counts are dependent variables, measured after the neural data, not independent variables that could plausibly cause the observed brain activity. The interpretive framing should reflect this directionality: the appropriate claim is that BOLD activity covaries with memory quality, not that memory quality predicts activity. A related issue concerns predictor collinearity: the behavioral results already show that detail counts depend on valence, meaning that valence and detail counts are not independent in the model. The authors should discuss what unique variance each predictor accounts for in the presence of the other and consider whether partial or orthogonalized predictors would clarify the interpretation. Finally, some result descriptions require revision to avoid implying directionality that the design does not support.

5. Recall repetition and memory trace transformation.

Each video was retrieved three times within the same session. A well-established literature demonstrates that repeated active retrieval modifies the memory trace, promoting abstraction, schematization, and pattern completion over successive recalls. The recall-recall RSA is framed primarily as an index of stability, but a more theoretically interesting question may be whether the representation changes systematically across rounds. For instance, whether emotional or perceptual features are differentially preserved or transformed. The authors should situate their stability measure within this literature and, where possible, examine whether representational change (rather than simply similarity) across recall rounds varies as a function of valence or detail type. Additionally, the basic finding that same-video patterns are more similar across recall repetitions than different-video patterns should be better framed as a validity check for the measure than as a substantive finding in its own right.

6. Conceptual validity of the template phase as an encoding proxy.

The template phase required participants to view ten sequential static frames from each video while the cue word was displayed, a procedure explicitly designed to reinforce the video-cue associations. This means that viewing the template stimuli likely engaged retrieval of the original encoding experience, not merely perceptual processing of static images. As a consequence, the template-recall RSA does not straightforwardly measure encoding-to-retrieval reactivation; it more accurately measures retrieval-to-retrieval pattern similarity, which is conceptually closer to the recall-recall RSA. The authors should address this confound explicitly and discuss whether the template phase can be considered a valid index of the representational content established at encoding. A consistent terminological convention should be adopted, with reactivation reserved for its technically precise meaning (reinstatement of an encoding-phase representation) or replaced with a more neutral term.

7. Pattern of null results and theoretical coherence.

Multiple predictions derived from prior literature were not confirmed: the relationship between medial temporal activity and perceptual detail was not modulated by negative valence; the dorsomedial subnetwork showed no stronger association with narrative detail for emotional memories; and no evidence was found for differential sensitivity to narrative versus perceptual recall along the hippocampal long axis. Individually, any one null result can be discussed as an informative boundary condition. Taken together, however, the pattern raises the question of whether the theoretical framework motivating the predictions requires revision, rather than whether each individual null result can be explained post hoc. The discussion should address this systematically. In particular, the absence of a significant association between any DMN subnetwork activity and narrative detail, arguably the most important null finding given the a priori prediction, receives only a brief treatment. This requires a more thorough discussion of what the null implies for accounts of DMN function in narrative memory retrieval, including consideration of whether the detail scoring operationalization (specific story events versus general schematic content) is partly responsible for the discrepancy. The authors should also reduce redundancy in the discussion: several passages restate findings from the Results without adding interpretive content.

8. Situating findings within the dimension-specificity framework.

The discussion situates the results within frameworks of DMN subnetwork specialization and emotional memory but does not engage with a relevant body of work on dimension-specific memory retrieval in the cued recollection literature. Studies using paradigms in which participants retrieve different types of event information within the same task have reported topographically distinct patterns of DMN engagement depending on the memory dimension targeted, with specialization concentrated in medial and lateral parietal nodes (e.g., Kwok et al., 2012, Human Brain Mapping; Procida et al., 2025, NeuroImage). The perceptual detail dimension in the present study maps naturally onto the detail/object dimension in this framework. The authors should discuss whether the subnetwork dissociations reported here are consistent with this account, and whether the contrast between perceptual and narrative content captures something qualitatively distinct from or continuous with the fractionation previously described in the cued recollection literature.

Minor Concerns

9. Overnight consolidation as a potential confound.

The delayed recall test was administered one day after scanning. Overnight consolidation processes are known to differentially benefit different types of memory content and could, in principle, have introduced a systematic dissociation between the neural activity measured during in-scanner recall and the behavioral measures used as predictors in the models. The authors should discuss this possibility in a limitations section (see below).

10. Trial ordering in the cued recall task.

The manuscript does not specify whether the order of cue presentation within each recall round was randomized across participants, fixed, or varied across rounds for the same participant. This information should be provided, as systematic ordering could introduce carry-over effects between valence conditions.

11. Run structure and integration of reappraisal trials.

The manuscript states that nine runs were completed in total (three rounds of three runs each), but does not clarify how the additional 12 reappraisal trials were distributed across the run structure. If they were interleaved within the same runs as standard recall trials, each run would have contained more trials than the current description implies; if they were in separate runs, the total run count is underreported. A precise description of the run structure, including total runs per participant and the distribution of reappraisal trials, is needed.

12. Head motion reporting.

The motion exclusion criterion (>20% of timepoints exceeding 0.30 mm framewise displacement) is stated, but no descriptive motion statistics are reported. The authors should provide mean framewise displacement values across participants and runs, the number of runs excluded due to excessive motion and for how many participants this occurred, and the average number of trials retained per valence condition after motion-related exclusions.

13. Incomplete reporting of pairwise comparisons.

For the overall correct details ANOVA, only the significant post-hoc comparison is reported. All pairwise comparisons, including non-significant ones, should be provided for completeness and transparency.

14. Asymmetric modeling across RSA approaches and handling of repeated recall trials.

Two asymmetries in the RSA analysis plan require clarification. First, the recall-recall stability analysis uses a primary model with narrative and perceptual details as the only predictors, with valence added in a secondary model; by contrast, the template-recall RSA appears to use the full model, including valence, from the outset, as implied by the parallel to the univariate approach. The rationale for this asymmetry should be stated explicitly, and consideration should be given to whether the primary analyses should be harmonized across the two RSA approaches. Second, for the template-recall RSA, the manuscript notes that event-specific reactivation was computed separately for each of the three recall trials associated with each video, but does not specify how these values were subsequently handled in the regression model. Were they averaged per video per participant, or entered as separate trials? This should be clarified.

15. Absence of a limitations section.

The manuscript does not include a dedicated limitations section. Given the several interpretive constraints identified above, a concise limitations paragraph should be added to the discussion

## Author Response

We thank the reviewers and editor for their feedback on our manuscript. We were encouraged that the reviewers regarded our research as well-motivated and conceptually meaningful (R2). We have revised the manuscript to address these comments, which included providing methodological context and theoretical clarification where needed. We believe these revisions have strengthened our paper and improved the clarity of the methods.

Major Concerns 1. Scoring protocol and inter-rater reliability.

The manuscript attributes the scoring protocol to Levine et al. (2002), but the category labels used here do not map directly onto the original Autobiographical Interview (AI) categories. If the protocol represents a modified version of the AI, the authors should state this explicitly and specify which categories were adapted, renamed, or newly introduced relative to the original. More critically, the manuscript provides no inter-rater reliability statistics anywhere, even though the central neuroimaging findings depend on the validity of these hand-scored behavioral measures. The authors should report intraclass correlation coefficients (ICCs) for all detail categories fed into the analyses (event, visual, and total correct) and describe how scoring disagreements were resolved. Finally, the manuscript states that several AI categories were excluded from analyses due to insufficient detail counts, but no descriptive statistics are provided for these excluded categories. Mean detail counts (with standard deviations) across videos and participants should be reported for all scored categories, both to document the scoring burden and to justify the exclusions.

RESPONSE: We have revised the manuscript to clarify that our scoring protocol was derived from the original Autobiographical Interview framework, rather than a direct implementation. Specifically, the AI's "perceptual" detail category was adapted to focus exclusively on visual-perceptual details and was referred to during scoring as "visual details". This modification was motivated by prior work demonstrating strong association between emotional memory and visual detail recollection (e.g., Kensinger et al., 2006, Journal of Memory and Language), whereas considerably less is known about other perceptual modalities such as auditory details. We now explicitly describe this adaptation in the Methods section: "belonging to one of the following categories, using a scheme derived from the Autobiographical Interview scoring protocol" (page 9) "'visual' (purely visual perceptual aspects not integral to the storyline)" (page 9) We have also added information regarding scoring procedures. To clarify, two independent raters scored a subset of the data to establish consistency in the scoring protocol. Then, one rater scored the remaining responses, all under supervision of the lead graduate student on the project. Because this study is a re-analysis of a dataset collected by a previous graduate student, these procedural details were inadvertently omitted from the original manuscript, and we apologize for the oversight.

To quantify scoring reliability, we calculated inter-rater reliability for the detail categories used here on a subset scored by both raters. Pearson correlations for each were as follows: Correct Details r = 0.92, Narrative details r = .83, Perceptual details = .93. This high level of agreement provides confidence in the reliability of the scoring procedure. "Two raters were trained on detail scoring until they achieved high reliability (correct detail inter-rater correlation = 0.92, narrative = .83, perceptual = .93); remaining datasets were scored by one of these raters under the supervision of a graduate student." (page 10) Lastly, we have specified in the scoring description that the mean number of details in each excluded category was fewer than one detail per recall, suggesting insufficient variability for meaningful statistical analysis. "All other scored categories did not yield high enough detail counts for analysis and thus were unsuitable for inclusion in the present study (category means across participants < 1 detail)." (page 10) 2. Operationalization of emotional valence.

Emotional valence was entered into the neuroimaging models as a categorical variable derived from the normative stimulus classifications in Samide et al. (2020), rather than from participants' own trial-wise ratings collected at both encoding and delayed recall. Given that affective responses to naturalistic stimuli vary substantially across individuals, this choice conflates stimulus category with subjective emotional experience, potentially attenuating the association between neural activity and the emotional tone of the memory as actually experienced. The authors should: (i) justify the preference for normative categorical valence over continuous subjective ratings as the primary neuroimaging predictor; (ii) report the correspondence between normative category assignments and individual participants' valence ratings; and (iii) examine whether substituting or supplementing the categorical predictor with trial-wise subjective ratings changes the pattern of results.

RESPONSE:

To assess the relationship between the normative valence categories and participants' subjective ratings at encoding, we examined participants' own trial-wise valence ratings at encoding (1 = most negative, 9 = most positive) as a function of the three valence categories. We have added the following text to the Design section of the Methods (page 7): "Mean valence ratings differed significantly across normative categories, F(2, 917) = 837.30, p < .001, all Tukey posthoc comparisons p <.001, with ratings increasing from negative (M = 2.85, SD = 1.54) to neutral (M = 5.95, SD = 1.35) to positive (M = 7.45, SD = 1.30) (Figure 1d)." The high correspondence between categories and subjective ratings seen here suggests that the categories capture the dominant dimension of participants' emotional experiences. The categorical approach balances trial counts across valence conditions and allows us to make interpretable contrasts (e.g., negative vs neutral, negative vs. positive) that align directly with our research questions and how valenced events are often situated in the literature. While continuous subjective ratings are a viable alternative, interpreting a 1-9 valence scale as a neuroimaging predictor introduces further ambiguity, such as how to define meaningful relational distance between ratings or modeling a nonlinear relationship between valence and emotional intensity. For these reasons, and given the strong alignment between normative categories and individual subjective ratings shown here, we have retained the original categorical approach.

We have included the above visualization to Figure 1 (see Figure 1c) to demonstrate the spread of the participants' average valence ratings across the three normative valence categories. While one participant showed less correspondence to the general pattern, tending to select a rating of "5" across categories, the remainder of the ratings appear to follow the pattern described above. The pattern of that participant can be seen in the plot below (not included in the manuscript).

3. Handling of forgotten and reappraisal trials in the GLM.

The manuscript states that analyses were restricted to remembered trials and that the 12 negative reappraisal trials were excluded from the present analyses. However, it is not stated whether forgotten trials and reappraisal trials were explicitly modeled as regressors of no interest in the first-level GLM. If they were simply omitted, their associated neural signal would remain in the residual time series and could contaminate the activity estimates for trials of interest. The authors should clarify how these trial types were handled at the GLM level and confirm whether their exclusion from the behavioral analyses was matched by appropriate nuisance modeling in the neuroimaging pipeline.

RESPONSE: We appreciate the reviewer's concern. We want to clarify that the present analyses used a single-trial analysis approach based on residualized fMRI time series data. Specifically, the first-level SPM12 model contained only nuisance regressors (e.g., motion parameters). The residual time series from this model were then extracted for each trial (which was possible due to our slow event-related design, where each trial spanned 15s) and used in linear mixed-effects modeling. Thus, the presence of forgotten trials and reappraisal trials would not affect the neural signal, because no task regressors were included in the first-level model. The exclusion of forgotten trials and reappraisal trials happened at the analysis stage after obtaining time-series data, at which point only remembered non-reappraisal trials were retained and used in the statistical models reported in the manuscript. We have added some wording to clarify this analysis approach in the Methods section (Preprocessing and Modeling of fMRI Data, page 13-14): "For the fMRI analyses, timeseries-averaged trial activity in each network was predicted using linear mixed-effects models,..." "...our mixed-effects analyses included only trials classified as "remembered" as indicated by participant rating of recall success at delayed retrieval" 4. Causal direction and predictor independence in the neuroimaging models.

The regression models treat the number of narrative and perceptual details recalled one day after scanning as predictors of BOLD activity acquired the previous day. This inverts the temporal and causal relationship: memory detail counts are dependent variables, measured after the neural data, not independent variables that could plausibly cause the observed brain activity. The interpretive framing should reflect this directionality: the appropriate claim is that BOLD activity covaries with memory quality, not that memory quality predicts activity. A related issue concerns predictor collinearity: the behavioral results already show that detail counts depend on valence, meaning that valence and detail counts are not independent in the model. The authors should discuss what unique variance each predictor accounts for in the presence of the other and consider whether partial or orthogonalized predictors would clarify the interpretation. Finally, some result descriptions require revision to avoid implying directionality that the design does not support.

RESPONSE: We have changed various wordings throughout the results to avoid using the word "predicts" when characterizing the relationship between BOLD activity and memory quality (Results, pages 18-20).

To assess collinearity between detail counts and valence category in our mixed-effects models, we used the R package car to compute generalized variance inflation factors (GVIF) for each model term in the basic activity model. Specifically, we use the adjusted statistic GVIF^(1/(2*Df)) due to multiple factors. Results indicated that perceptual details (GVIF_adj = 1.52), valenceCategory (GVIF_adj = 1.03), and both interaction terms (GVIF_adj = 1.24 and 1.42) were well within acceptable limits (GVIF_adj < 2.24 or VIF > 5). Narrative details showed a GVIF_adj of 2.00, indicating greater predictor overlap than for the other terms but falling below the threshold typically considered indicative of a serious collinearity problem (Fox &Monette, 1992; O'Brien, 2007). Because our mixed-effects regression approach inherently estimates each variable's effect while holding all others constant, the model already accounts for shared variance between variables (such as narrative details and valence). We therefore consider these GVIF results acceptable without need for orthogonalization.

5. Recall repetition and memory trace transformation.

Each video was retrieved three times within the same session. A well-established literature demonstrates that repeated active retrieval modifies the memory trace, promoting abstraction, schematization, and pattern completion over successive recalls. The recall-recall RSA is framed primarily as an index of stability, but a more theoretically interesting question may be whether the representation changes systematically across rounds. For instance, whether emotional or perceptual features are differentially preserved or transformed. The authors should situate their stability measure within this literature and, where possible, examine whether representational change (rather than simply similarity) across recall rounds varies as a function of valence or detail type. Additionally, the basic finding that same-video patterns are more similar across recall repetitions than different-video patterns should be better framed as a validity check for the measure than as a substantive finding in its own right.

RESPONSE: We have now included a caveat in our discussion as follows: "While this measure informs the consistency of the representation across repeated retrievals, we did not directly examine the degree of representational change across the individual recall rounds. The current study was limited in answering this question because there were relatively few trials per round and because the effects of repeated retrieval were confounded with the effects of time. Future studies could resolve these issues to determine whether different memory features are differentially transformed with repeated retrieval." (page 29) 6. Conceptual validity of the template phase as an encoding proxy.

The template phase required participants to view ten sequential static frames from each video while the cue word was displayed, a procedure explicitly designed to reinforce the video-cue associations. This means that viewing the template stimuli likely engaged retrieval of the original encoding experience, not merely perceptual processing of static images. As a consequence, the template-recall RSA does not straightforwardly measure encoding-to-retrieval reactivation; it more accurately measures retrieval-to-retrieval pattern similarity, which is conceptually closer to the recall-recall RSA. The authors should address this confound explicitly and discuss whether the template phase can be considered a valid index of the representational content established at encoding. A consistent terminological convention should be adopted, with reactivation reserved for its technically precise meaning (reinstatement of an encoding-phase representation) or replaced with a more neutral term.

RESPONSE: We have further included description of the template phase in the discussion: "It should be acknowledged that template-phase patterns should not be considered as a straightforward measure of encoding-related activity, but rather a high-fidelity representation of what was previously encoded, incorporating both perceived and remembered information. Thus, template-recall similarity reflects how similar the recall patterns were to an event-specific instance of highly detailed retrieval. The current findings suggest that recall activity patterns may better capture memory quality than the template phase, where other non-remembered perceptual features may influence patterns more strongly, making template-recall similarity less sensitive to memory details that are preserved over time." (page 29) We have also added clarification about our interpretation in the Methods: "We reasoned that this measure of template-recall similarity would be sensitive to the reactivation of high-fidelity, event-specific details from encoding that span the template and recall phases." (page 21) 7. Pattern of null results and theoretical coherence.

Multiple predictions derived from prior literature were not confirmed: the relationship between medial temporal activity and perceptual detail was not modulated by negative valence; the dorsomedial subnetwork showed no stronger association with narrative detail for emotional memories; and no evidence was found for differential sensitivity to narrative versus perceptual recall along the hippocampal long axis. Individually, any one null result can be discussed as an informative boundary condition. Taken together, however, the pattern raises the question of whether the theoretical framework motivating the predictions requires revision, rather than whether each individual null result can be explained post hoc. The discussion should address this systematically. In particular, the absence of a significant association between any DMN subnetwork activity and narrative detail, arguably the most important null finding given the a priori prediction, receives only a brief treatment. This requires a more thorough discussion of what the null implies for accounts of DMN function in narrative memory retrieval, including consideration of whether the detail scoring operationalization (specific story events versus general schematic content) is partly responsible for the discrepancy. The authors should also reduce redundancy in the discussion: several passages restate findings from the Results without adding interpretive content.

RESPONSE: We have revised our Discussion to reduce redundancy when possible. We acknowledge that many of the results did not turn out as originally expected. However, we are hesitant to revise our theoretical framework based on the null results observed here. While null results may reflect meaningful deviations from theoretical predictions, they may also reflect idiosyncratic features of an individual study, such as the sample characteristics or specific experimental design choices. We highlight below some discussion of the boundary conditions of our theoretical framework that might account for the current results: "Surprisingly, DMN activity did not relate to the amount of narrative detail recalled, diverging from previous findings implicating lateral temporal and medial frontal regions in narrative retrieval (Baetens et al., 2014; Binder et al., 2009; Gurguryan &Sheldon, 2019). Although we had hypothesized that narrative detail recall would correspond to dorsomedial and core midline activation, there are a number of reasons why the current paradigm may have produced different results. It is possible that the types of narrative details supported by the dorsomedial subnetwork were not captured in participants' written recalls, as our measure focused on specific story details rather than general conceptual or schematic information, which might rely more strongly on frontal and temporal areas." (page 27) "If emotion was primarily conveyed through narrative, rather than perceptual details, this may also explain the finding that valence modulated the relationship between narrative detail and template-recall similarity in the medial temporal subnetwork, with a stronger relationship for negative compared to positive stimuli. This unexpected finding suggests that high-fidelity memory reinstatement in these areas may be involved in prioritizing or maintaining narrative details for naturalistic negative events. This pattern of results also points to the importance of considering which kinds of details carry the emotional impact of the event." (page 25) "The current findings suggest that recall activity patterns may better capture memory quality than the template phase, where other non-remembered perceptual features may influence patterns more strongly, making template-recall similarity less sensitive to memory details that are preserved over time." (page 29) 8. Situating findings within the dimension-specificity framework.

The discussion situates the results within frameworks of DMN subnetwork specialization and emotional memory but does not engage with a relevant body of work on dimension-specific memory retrieval in the cued recollection literature. Studies using paradigms in which participants retrieve different types of event information within the same task have reported topographically distinct patterns of DMN engagement depending on the memory dimension targeted, with specialization concentrated in medial and lateral parietal nodes (e.g., Kwok et al., 2012, Human Brain Mapping; Procida et al., 2025, NeuroImage). The perceptual detail dimension in the present study maps naturally onto the detail/object dimension in this framework. The authors should discuss whether the subnetwork dissociations reported here are consistent with this account, and whether the contrast between perceptual and narrative content captures something qualitatively distinct from or continuous with the fractionation previously described in the cued recollection literature.

RESPONSE: We have included the Procida et al. (2025) paper in our Discussion: "Here, we find that such differences extend to contents of episodic memory: whereas perceptual details may contribute to the episodic specificity of memory, valence may be tied to more abstract (i.e., less detailed) event representations. This pattern complements existing research showing that DMN engagement varies depending on the type of information being recalled, such as objects, characters, or places (Procida et al. 2025; Gilmore et al., 2021; Ritchey, 2026)." (page 27) Minor Concerns 9. Overnight consolidation as a potential confound.

The delayed recall test was administered one day after scanning. Overnight consolidation processes are known to differentially benefit different types of memory content and could, in principle, have introduced a systematic dissociation between the neural activity measured during in-scanner recall and the behavioral measures used as predictors in the models. The authors should discuss this possibility in a limitations section (see below).

RESPONSE: We have updated our discussion to acknowledge this possibility: "However, there may have been additional details recalled during the scan that were forgotten during delayed recall, which would not be captured by our analysis approach. For example, consolidation processes occurring between the two tests could have differentially impacted certain types of details, for instance, weakening perceptual details while strengthening semantic gist (Robin &Moscovitch, 2017; Lifanov et al., 2021). Future research incorporating immediate tests and separate measures for general and specific memory content will help clarify how brain activity relates to the persistence of memory details across time." (page 28) 10. Trial ordering in the cued recall task.

The manuscript does not specify whether the order of cue presentation within each recall round was randomized across participants, fixed, or varied across rounds for the same participant. This information should be provided, as systematic ordering could introduce carry-over effects between valence conditions.

RESPONSE: The order of cue presentation during the cued recall task was randomized for each participant and further randomized across each of the three recall rounds, minimizing the possibility of order-related carry-over effects. To specify this, we have added the following text to the Cued Recall Task section of the Methods: "The order of video presentation was randomized across participants and across rounds for each participant." (page 8) 11. Run structure and integration of reappraisal trials.

The manuscript states that nine runs were completed in total (three rounds of three runs each), but does not clarify how the additional 12 reappraisal trials were distributed across the run structure. If they were interleaved within the same runs as standard recall trials, each run would have contained more trials than the current description implies; if they were in separate runs, the total run count is underreported. A precise description of the run structure, including total runs per participant and the distribution of reappraisal trials, is needed.

RESPONSE:

The additional reappraisal trials were interleaved within the same nine runs as the standard recall trials, rather than presented in separate runs. Each run contained both trial types, with 4 reappraisal trials added per run (12 reappraisal trials per round, 36 total across the task), on top of the previously described recall trials. The total run count remains nine runs (three rounds of three runs each); the per-run trial count has now been corrected to reflect the inclusion of these additional trials (though they are not part of our analysis). We have updated the Methods section to specify that each run contained 4 additional reappraisal trials: "Note that, for each participant, an additional 12 negative videos were recalled under instructions to reappraise the video during the elaboration period (4 additional trials per run)". (page 8) 12. Head motion reporting.

The motion exclusion criterion (>20% of timepoints exceeding 0.30 mm framewise displacement) is stated, but no descriptive motion statistics are reported. The authors should provide mean framewise displacement values across participants and runs, the number of runs excluded due to excessive motion and for how many participants this occurred, and the average number of trials retained per valence condition after motion-related exclusions.

RESPONSE:

Five total participants were excluded, which has now been specified in the Methods section. As described in the manuscript, three participants were excluded due to insufficient usable data (e.g., lack of behavioral data, or exiting the scanner early). Two further participants had at least three runs excluded because they exceeded our predefined motion criterion, and so they were excluded at the participant level. Lastly, five runs across three participants were excluded using the motion exclusion criterion, which has now been added to in the Methods section: "Scan runs were excluded from analyses if >20% of time points exceeded a framewise displacement of 0.30 mm. Two participants were excluded because three of their scan runs met this criterion (final N = 30). An additional 3 participants were retained but had 1 or 2 of their runs excluded based on this criterion." (page 11) The average number of trials retained per valence condition after motion related exclusions and after relevant trial exclusions (remembered only) are now provided in the manuscript: "After all scan and trial exclusions, analyses were based on the following trial counts per participant: positive M = 32 (SD = 4.43); neutral M = 32.333 (SD = 3.96); negative M = 29 (SD = 5.445)." (page 11-12) 13. Incomplete reporting of pairwise comparisons.

For the overall correct details ANOVA, only the significant post-hoc comparison is reported. All pairwise comparisons, including non-significant ones, should be provided for completeness and transparency.

RESPONSE: We have now added non-significant pairwise post-hoc comparisons for the correct details ANOVA, as well as the other two ANOVAs reported in that section. (see page 17) 14. Asymmetric modeling across RSA approaches and handling of repeated recall trials.

Two asymmetries in the RSA analysis plan require clarification. First, the recall-recall stability analysis uses a primary model with narrative and perceptual details as the only predictors, with valence added in a secondary model; by contrast, the template-recall RSA appears to use the full model, including valence, from the outset, as implied by the parallel to the univariate approach. The rationale for this asymmetry should be stated explicitly, and consideration should be given to whether the primary analyses should be harmonized across the two RSA approaches. Second, for the template-recall RSA, the manuscript notes that event-specific reactivation was computed separately for each of the three recall trials associated with each video, but does not specify how these values were subsequently handled in the regression model. Were they averaged per video per participant, or entered as separate trials? This should be clarified.

RESPONSE: We have now resolved the inconsistency in how we describe our modeling approach for the two RSAs, and have updated them to be clearer and more consistent. See Methods section Statistical Analysis: Representational Similarity Analysis (page 15), and Results section Event-specific patterns of activation and their relation to memory quality (page 22).

15. Absence of a limitations section.

The manuscript does not include a dedicated limitations section. Given the several interpretive constraints identified above, a concise limitations paragraph should be added to the discussion RESPONSE: We have addressed the interpretive constraints throughout the discussion, presenting them alongside relevant findings but not grouping them into a separate paragraph. We believe this approach may better contextualize these limitations.
