# Supplementary material for: Remembered Event Features Shape Default Mode Network Engagement during Emotional Memory Recall
Source: eNeuro. 2026 Nov 6;13(11):ENEURO.0146-26.2026. doi: 10.1523/ENEURO.0146-26.2026 (PMC13750827; doi:10.1523/ENEURO.0146-26.2026)
Supplement: Supplementary file 1 [file eneuro-13-ENEURO.0146-26.2026-s001.docx]

| **Term** | **Network** | **Region label** | **Hemi** | **Estimate** | **df** | **t** | **p (FDR)** |
| --- | --- | --- | --- | --- | --- | --- | --- |
| Negative valence | ContA | IPS_1 | LH | 0.1380 | 2,720 | 3.25 | p < .01 |
|  | ContA | IPS_1 | RH | 0.1480 | 2,720 | 3.75 | p < .01 |
|  | ContA | PFCl_1 | LH | 0.1200 | 2,720 | 4.62 | p < .001 |
|  | ContA | PFCl_2 | RH | 0.1360 | 2,730 | 6.37 | p < .001 |
|  | ContA | PFCl_3 | LH | 0.0661 | 2,720 | 2.75 | p < .05 |
|  | ContB | IPL_1 | RH | 0.2450 | 2,720 | 4.66 | p < .001 |
|  | ContB | PFCl_1 | LH | 0.1140 | 2,720 | 3.37 | p < .01 |
|  | ContB | PFCld_2 | RH | 0.1160 | 2,720 | 3.92 | p < .01 |
|  | ContB | PFClv_2 | RH | 0.0875 | 2,720 | 2.63 | p < .05 |
|  | ContB | PFCmp_1 | RH | 0.1030 | 2,720 | 4.06 | p < .001 |
|  | ContB | Temp_1 | RH | 0.0562 | 2,720 | 3.84 | p < .01 |
|  | ContC | pCun_1 | LH | 0.1100 | 2,720 | 4.07 | p < .001 |
|  | ContC | pCun_1 | RH | 0.1200 | 2,720 | 4.35 | p < .001 |
|  | ContC | pCun_2 | RH | 0.1240 | 2,720 | 4.94 | p < .001 |
|  | DefaultA | IPL_1 | LH | 0.1040 | 2,720 | 2.65 | p < .05 |
|  | DefaultA | IPL_1 | RH | 0.1160 | 2,720 | 4.54 | p < .001 |
|  | DefaultA | PFCm_1 | LH | -0.0394 | 2,720 | -3.23 | p < .01 |
|  | DefaultA | pCunPCC_1 | LH | 0.0735 | 2,720 | 5.11 | p < .001 |
|  | DefaultA | pCunPCC_1 | RH | 0.0878 | 2,720 | 5.77 | p < .001 |
|  | DefaultA | pCunPCC_3 | LH | 0.0573 | 2,720 | 3.82 | p < .01 |
|  | DefaultB | AntTemp_1 | RH | 0.0608 | 2,720 | 4.48 | p < .001 |
|  | DefaultB | IPL_1 | LH | 0.1100 | 2,720 | 3.99 | p < .001 |
|  | DefaultB | PFCd_1 | RH | 0.1280 | 2,720 | 4.45 | p < .001 |
|  | DefaultB | PFCd_3 | LH | 0.0714 | 2,720 | 3.44 | p < .01 |
|  | DefaultB | PFCv_1 | LH | 0.0499 | 2,720 | 2.99 | p < .05 |
|  | DefaultB | PFCv_1 | RH | 0.1100 | 2,720 | 4.70 | p < .001 |
|  | DefaultB | PFCv_2 | LH | -0.0504 | 2,720 | -2.93 | p < .05 |
|  | DefaultB | PFCv_3 | LH | 0.0978 | 2,720 | 2.91 | p < .05 |
|  | DefaultB | PFCv_4 | LH | 0.0773 | 2,720 | 3.17 | p < .05 |
|  | DefaultB | Temp_1 | RH | 0.1600 | 2,720 | 6.10 | p < .001 |
|  | DefaultB | Temp_4 | LH | 0.0871 | 2,720 | 3.29 | p < .01 |
|  | DefaultC | IPL_1 | RH | 0.0677 | 2,720 | 2.63 | p < .05 |
|  | DorsAttnA | SPL_2 | RH | 0.1310 | 2,720 | 2.74 | p < .05 |
|  | LimbicA | TempPole_2 | RH | 0.0310 | 2,730 | 3.19 | p < .05 |
|  | LimbicB | OFC_1 | RH | 0.0275 | 2,740 | 2.65 | p < .05 |
|  | SalVentAttnA | FrOper_1 | LH | -0.0304 | 2,720 | -2.71 | p < .05 |
|  | SalVentAttnA | ParOper_1 | LH | -0.0921 | 2,720 | -3.32 | p < .01 |
|  | SalVentAttnA | ParOper_1 | RH | -0.0769 | 2,730 | -2.58 | p < .05 |
|  | TempPar | 2 | LH | 0.0638 | 2,720 | 2.62 | p < .05 |
|  | VisCent | ExStr_1 | LH | 0.0528 | 2,720 | 2.68 | p < .05 |
|  | VisCent | ExStr_2 | LH | 0.2000 | 2,720 | 4.46 | p < .001 |
|  | VisCent | ExStr_3 | RH | 0.1420 | 2,720 | 3.46 | p < .01 |
|  | VisCent | Striate_1 | RH | 0.0939 | 2,720 | 2.60 | p < .05 |
| Positive valence | ContB | Temp_1 | RH | 0.0643 | 2,720 | 4.48 | p < .001 |
|  | ContC | Cingp_1 | RH | 0.0397 | 2,720 | 2.98 | p < .05 |
|  | ContC | pCun_2 | RH | 0.1210 | 2,720 | 4.94 | p < .001 |
|  | DefaultA | IPL_1 | RH | 0.0795 | 2,720 | 3.17 | p < .05 |
|  | DefaultA | pCunPCC_1 | LH | 0.0684 | 2,720 | 4.85 | p < .001 |
|  | DefaultA | pCunPCC_1 | RH | 0.0858 | 2,720 | 5.75 | p < .001 |
|  | DefaultA | pCunPCC_3 | LH | 0.0664 | 2,720 | 4.52 | p < .001 |
|  | DefaultB | AntTemp_1 | RH | 0.0531 | 2,720 | 3.99 | p < .01 |
|  | DefaultB | PFCd_1 | RH | 0.0983 | 2,720 | 3.48 | p < .05 |
|  | DefaultB | Temp_1 | RH | 0.0931 | 2,720 | 3.62 | p < .01 |
|  | DefaultC | PHC_1 | LH | -0.0245 | 2,720 | -3.48 | p < .05 |
|  | SalVentAttnA | Ins_1 | RH | 0.0313 | 2,730 | 3.07 | p < .05 |
|  | TempPar | 2 | LH | 0.1070 | 2,720 | 4.48 | p < .001 |

***Extended Data Table 1-1.*** Significant associations between parcel activity and predictors across the whole brain, using a threshold of FDR-corrected p < .05. Here, DefaultA corresponds to core midline, DefaultB to dorsomedial subnetwork, and DefaultC to medial temporal subnetwork. Network and region labels are from the Schaefer atlas. LH=left hemisphere, RH=right hemisphere.
