# Supplementary material for: Remembered Event Features Shape Default Mode Network Engagement during Emotional Memory Recall
Source: eNeuro. 2026 Nov 6;13(11):ENEURO.0146-26.2026. doi: 10.1523/ENEURO.0146-26.2026 (PMC13750827; doi:10.1523/ENEURO.0146-26.2026)
Supplement: Supplementary file 2 [file eneuro-13-ENEURO.0146-26.2026-s002.docx]

Template-recall reactivation

| **Term** | **Network** | **Region label** | **Hemi** | **Estimate** | **df** | **t** | **p (FDR)** |
| --- | --- | --- | --- | --- | --- | --- | --- |
| Positive valence | DefaultC | PHC_1 | RH | 0.01140 | 2,780 | 4.06 | p < .05 |
| Narrative details × Negative valence | VisCent | ExStr_3 | LH | 0.01560 | 2,790 | 3.84 | p < .05 |
| Perceptual details × Positive valence | VisCent | ExStr_5 | RH | 0.01180 | 2,790 | 3.44 | p < .05 |
|  | VisPeri | ExStrInf_2 | LH | 0.01150 | 2,790 | 4.82 | p < .001 |
|  | VisPeri | ExStrSup_1 | RH | 0.00963 | 2,790 | 3.36 | p < .05 |
|  | VisPeri | StriCal_1 | RH | 0.01350 | 2,790 | 4.57 | p < .001 |

Recall stability

| **Term** | **Network** | **Region label** | **Hemi** | **Estimate** | **df** | **t** | **p (FDR)** |
| --- | --- | --- | --- | --- | --- | --- | --- |
| Perceptual details | DefaultB | PFCv_2 | LH | 0.00658 | 2,770 | 4.70 | p < .001 |
|  | DorsAttnB | PostC_4 | RH | 0.00920 | 2,780 | 3.39 | p < .05 |
|  | SomMotA | 9 | RH | 0.00931 | 2,790 | 4.07 | p < .01 |
| Positive valence | ContA | IPS_2 | LH | 0.02920 | 2,770 | 3.64 | p < .05 |
|  | ContB | IPL_1 | RH | 0.03820 | 2,770 | 3.53 | p < .05 |
|  | ContB | PFCld_1 | RH | 0.02370 | 2,770 | 3.07 | p < .05 |
|  | ContB | PFCld_2 | RH | 0.02950 | 2,770 | 3.61 | p < .05 |
|  | ContB | PFClv_2 | RH | 0.02650 | 2,770 | 3.20 | p < .05 |
|  | DefaultA | PFCd_1 | RH | 0.01610 | 2,770 | 3.29 | p < .05 |
|  | DefaultA | PFCm_2 | LH | 0.01930 | 2,770 | 2.95 | p < .05 |
|  | DefaultB | PFCd_1 | RH | 0.03370 | 2,770 | 4.70 | p < .001 |
|  | DefaultB | PFCd_3 | LH | 0.02210 | 2,770 | 3.95 | p < .01 |
|  | DefaultB | PFCv_2 | LH | 0.02230 | 2,770 | 5.05 | p < .001 |
|  | LimbicB | OFC_1 | RH | 0.01090 | 2,770 | 3.54 | p < .05 |
|  | LimbicB | OFC_2 | RH | 0.01040 | 2,770 | 3.22 | p < .05 |
|  | LimbicB | OFC_4 | RH | 0.01820 | 2,770 | 3.01 | p < .05 |
|  | VisCent | ExStr_3 | RH | 0.02320 | 2,780 | 2.96 | p < .05 |
| Narrative details × Negative valence | VisCent | ExStr_3 | LH | 0.01750 | 2,750 | 3.85 | p < .05 |
| Perceptual details × Negative valence | VisPeri | ExStrSup_3 | RH | 0.02170 | 2,790 | 4.37 | p < .01 |
| Perceptual details × Positive valence | ContC | Cingp_1 | RH | -0.00596 | 2,790 | -3.55 | p < .05 |
|  | DorsAttnA | SPL_1 | RH | 0.01150 | 2,790 | 3.52 | p < .05 |

***Extended Data Table 2-1***. Significant associations between our two RSA measures and predictors across the whole brain, using a threshold of FDR-corrected *p* < .05. Here, DefaultA corresponds to core midline, DefaultB to dorsomedial subnetwork, and DefaultC to medial temporal subnetwork. Network and region labels are from the Schaefer atlas. LH=left hemisphere, RH=right hemisphere.
